# Individualized temporal patterns drive human sleep spindle timing

**DOI:** 10.1073/pnas.2405276121

**Published:** 2025-01-07

**Authors:** Shuqiang Chen, Mingjian He, Ritchie E. Brown, Uri T. Eden, Michael J. Prerau

**Affiliations:** ^a^Graduate Program for Neuroscience, Boston University, Boston, MA 02215; ^b^Department of Anesthesia, Critical Care and Pain Medicine, Massachusetts General Hospital, Boston, MA 02114; ^c^Harvard-MIT Health Sciences and Technology, Massachusetts Institute of Technology, Cambridge, MA 02139; ^d^Department of Psychiatry, Veterans Affairs Boston Healthcare System and Harvard Medical School, Boston, MA 02132; ^e^Department of Mathematics and Statistics, Boston University, Boston, MA 02215; ^f^Division of Sleep and Circadian Disorders, Brigham and Women’s Hospital, Boston, MA 02115; ^g^Department of Medicine, Harvard Medical School, Boston, MA 02115

**Keywords:** sleep spindles, timing patterns, slow oscillations, infraslow activity, point processes

## Abstract

Sleep spindles are cortical electrical oscillations considered critical for memory consolidation and sleep stability. The timing and pattern of sleep spindles are likely to be important in driving synaptic plasticity during sleep as well as preventing disruption of sleep by sensory and internal stimuli. However, the relative importance of factors such as sleep depth, cortical up/down-state, and temporal clustering in governing sleep spindle dynamics remains poorly understood. Here, we analyze sleep data from 1,025 participants, statistically modeling the simultaneous influences of multiple factors on moment-to-moment spindle production using a point process-generalized linear model framework. Results reveal fingerprint-like timing patterns, characterized by a refractory period followed by a period of increased spindle activity, which are highly individualized yet consistent night-to-night, with increased variability with age. Strikingly, short-term (<15 s) temporal patterns of past spindle history are the main determinant of spindle timing, accounting for over 70% of the statistical deviance—surpassing the contribution of factors such as cortical up/down-state (slow oscillation phase), sleep depth, and long-term history (15 to 90 s, including ~50 s infraslow activity). Short-term history has a statistically significant influence in over 98% of the population, suggesting it is a near-universal feature of spindle activity. Short-term history and slow oscillation phase exert independent effects on spindle timing. Our results establish a robust statistical framework to examine abnormalities in sleep spindle timing observed in neurological disorders and aging, as well as the relationship between individualized sleep spindle timing, cognition, and sleep stability.

## Significance

Sleep spindles are cortical electrical waveforms observed during sleep, considered critical for memory consolidation and sleep stability. Abnormalities in sleep spindles have been found in neuropsychiatric disorders and aging and are believed to contribute to functional deficits. Here, using a rigorous statistical framework, we demonstrate that short-term timing patterns are the dominant determinant of spindle timing, whereas sleep depth, cortical up/down-state, and long-term (infraslow) pattern, features thought to be primary drivers of spindle occurrence, are less important. We also show that these short-term timing patterns are fingerprint-like and show increased variability over age. This study provides an alternative lens on spindle production mechanisms, which will allow studies of the role of spindle timing patterns in memory consolidation, aging, and disease.

Sleep spindles, a subset (1, 2) of transient neural oscillatory bursts (typically lasting 0.5 to 1.5 s) during non-rapid eye movement (NREM) sleep, are visually observed as ~ 10 to 16 Hz fluctuating waveforms in the sleep electroencephalogram (EEG) in humans (3, 4). Since their initial discovery in 1935 (3), sleep spindles have been of great interest due to their association with memory consolidation (5), sleep stability (6, 7), neurological disorders such as Alzheimer’s disease, schizophrenia, epilepsy, and Parkinson’s disease (4), as well as noted changes during natural aging (8).

Numerous studies have shown that the amount of spindle activity evolves dynamically over time, influenced by factors such as sleep stage (4, 9), the phase of the slow oscillation (0.4 to 1.5 Hz) (4, 10), hippocampal ripples (11–13), and the activity of locus coeruleus neurons that control spindle infraslow activity (~0.02 Hz) (14, 15). More recently, studies have started to assess the temporal patterns of spindles, in particular, spindle refractoriness, spindle train clustering, and how they affect sleep-dependent memory consolidation and sleep stability (4, 16–19). The timing and pattern of sleep spindles are likely to be important in determining synaptic plasticity during sleep as well as preventing disruption of sleep by sensory and internal stimuli (4). However, a general framework which can account for the precise moment-to-moment regulation of spindle production, as well as the relative influences and interactions of these factors simultaneously, is lacking.

Thus, in this study, we characterize spindle dynamics across large, multiethnic, cross-sectional datasets of 1,025 human participants. For each participant, we quantify the influences and interactions of sleep stage, slow oscillation phase, and short and long-term (including infraslow) effects of past spindles on the moment-to-moment probability of spindle generation using a point process statistical modeling framework (20, 21). We then evaluate the results across the whole population to build a comprehensive picture of the different mechanisms simultaneously governing human spindle production, as well as the variability observed between individuals, across age and other demographic factors. Surprisingly, we find that short-term (<15 s), individualized, temporal patterns of past spindle history are the main determinant of spindle timing.

## 1. Results

### 1.1. Study Overview: Spindle Modeling and Population Studied.

To create an integrated approach to understanding the multiple factors governing spindle generation, we develop a model using a point process-generalized linear model (GLM) framework (20, 21). Point processes are mathematical models, which describe discrete events occurring in space or time. A *conditional intensity function* describes the instantaneous spindle density given the history of past events and allows us to quantify the relationship between influencing factors (including sleep stage/depth, slow oscillation phase, and the timing of previous spindles) and moment-to-moment spindle density (rate expressed in spindles/min). Using this model, we can estimate individualized parameters for each participant that represent how each factor contributes to that individual’s spindle rate. The model estimates provide useful information such as slow oscillation phase coupling preference and magnitude, the effect of sleep stage on the spindle rate, and the effect of a previous spindle on the current rate. Moreover, by comparing models with different combinations of factors in a large population, we can assess the relative contribution of different factors to the moment-to-moment production of spindles. Overall, this approach provides a robust statistical framework for characterizing the numerous interacting neurophysiological mechanisms underlying spindle production. We describe the model in the *Methods*, with further details provided in *SI Appendix*, Methods.

We fit this model to a dataset from Wamsley et al. (22) with two-night sleep EEG recordings that included 17 healthy control participants (age range: 26 to 45 y; age mean: 36.31 ± 7.12 y; M/F: 14/3), as well as the Multi-Ethnic Study of Atherosclerosis (MESA), a large cross-sectional dataset (23, 24) with single-night recordings for 1,008 participants. MESA participants were divided into a middle-aged group (433 participants, age range: 54 to 65 y, age mean: 60.31 ± 3.01 y, M/F: 228/205), and an older group (575 participants, age range: 66 to 94 y, age mean 75.09 ± 2.30 y, M/F: 295/280). For each polysomnography record, we identified time-frequency peaks (1) in the sigma range (12 to 16 Hz), aligning with the frequencies of traditional definitions of “fast” spindles and fit the models with their associated covariates (*SI Appendix*, Methods).

### 1.2. Integrating Information from Multiple Simultaneous Influences on Spindle Activity.

**Fig. 1.**
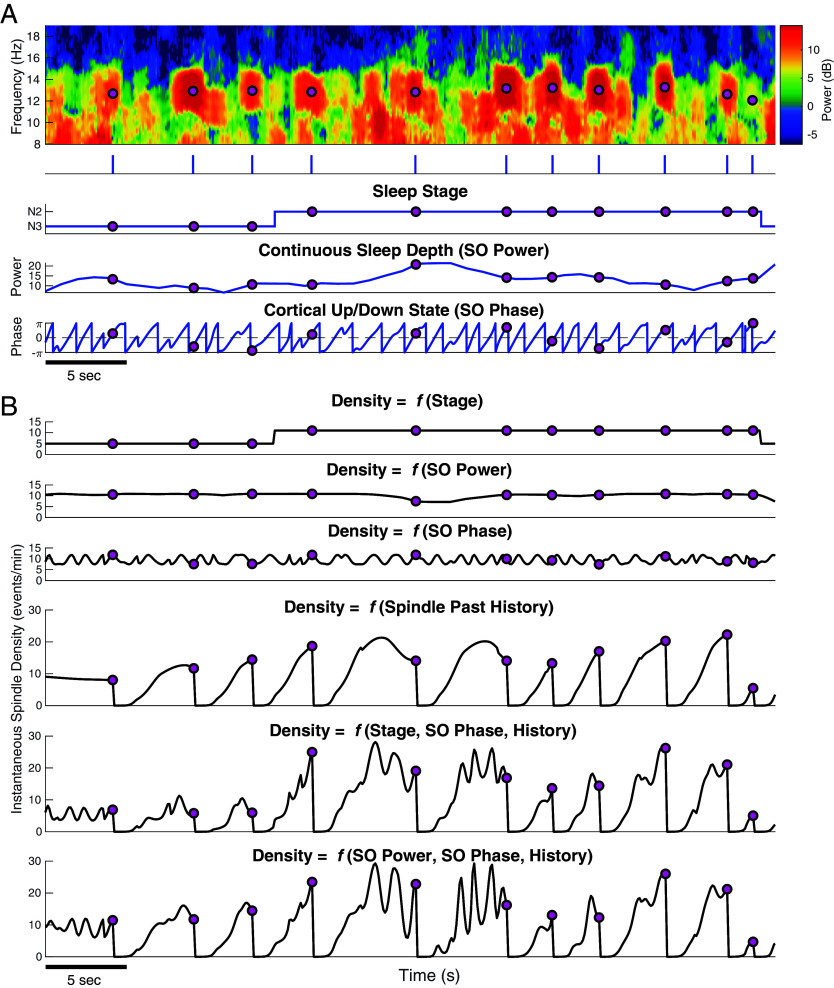
Integrating multiple simultaneous influences on spindle timing through a point process-GLM framework. (*A*) From *Top* to *Bottom*, we show the EEG multitaper spectrogram, spindle train, hypnogram, SO power, and SO phase, with pink circles indicating detected spindles. (*B*) Model estimates of instantaneous spindle density through single factors and multiple components with interactions. Results are shown for a single participant over a period of 45 s. Note the differences in the scales of instantaneous spindle density on the *y* axis for models with/without history.

In Fig. 1, we provide an exemplary overview of point process-GLMs on the individual level to assess the effect of each neurophysiological factor on spindle dynamics, as well as that of integrating multiple factors. Fig. 1*A* shows central EEG data from a single participant (Wamsley dataset) over 45 s, along with the associated factors used in this approach. Shown on top are the multitaper spectrogram (25) with detected spindles (pink circles), followed by the spindle train (times of detected spindles). Below, we show influencing factors including sleep stage (discrete sleep depth), slow oscillation (SO) power [a continuous metric of sleep depth (2, 26)], and SO phase (cortical up/down- states). Fig. 1*B* then shows instantaneous spindle density (conditional intensity) computed by modeling each of these factors, including spindle past history alone, and also estimates from models combining multiple features and their interactions. As the density is directly related to the probability of observing a spindle, a factor informative in spindle timing will produce an instantaneous density with high rates when spindles occur and low rates when there are no spindles (see estimates from models with history components in Fig. 1*B*). Conversely, an uninformative factor will have a rate unrelated to the observed spindle times (see model estimates for sleep stage, *Top* panel in Fig. 1*B*). We can therefore develop intuition about the information provided by each component by examining the way in which the instantaneous density changes with respect to the observed spindle times.

We first examine how sleep stage impacts spindle activity (Fig. 1 *B*, *Top*). In this sleep stage only model, we see the spindle density is a discrete, stepwise function that has the highest value in N2 (10.99 events/min) stage and goes down in the N3 stage (4.96 events/min). This model is mathematically equivalent to computing the average spindle density within each stage. The density is constant within a given stage, which imposes the assumption that spindles are equally likely at all times within the same stage. Using sleep stage alone therefore models spindle activity as a series of step-wise baseline rates, which describe the average activity but are not overly informative about individual spindle times.

We can switch from discrete stage to continuous sleep depth (second panel) by modeling slow oscillation (<1.5 Hz) power (SOP) (2). SO-power provides a more objective assessment of sleep depth compared to sleep stage, whose scoring criteria rely on the presence of spindles. Now, instead of step-wise constant rates, the instantaneous density shows continuous variation, although that variation is subtle on short time scales and does not clearly track spindle activity. However, it eliminates potentially unphysiological instantaneous jumps in rates at scored stage transitions, allowing for gradual transitions in spindle density with increasing/decreasing sleep depth. Thus, continuous sleep depth may be somewhat more informative than discrete sleep stage while remaining objective.

We next examine the factor of cortical up/down-state (third panel), as measured by the SO-phase. In this case, the density fluctuates between 7.5 and 11.8 events/min as a function of phase, indicating very specific short periods of higher and lower probability of spindle production, corresponding with the up and down-states, respectively. While many spindles occur close to the up-state of SO, consistent with many previous studies (10, 27, 28), many spindles also occur at other phases and during the down-state. Moreover, the vast majority of SO up-states occur without any spindle activity at all. Thus, it suggests that phase by itself may be informative during some periods, but uninformative or misleading at other times.

We then examine the impact of spindle event temporal patterns on spindle dynamics (fourth panel), in which the instantaneous density is a function of the history of previous spindle times. We will describe history and temporal patterns in detail in the next section. In contrast to the previous estimates, which fluctuate around a central nonzero density, we see that immediately after each spindle the density drops toward 0 and remains there through a period where no events will occur, indicating a refractory period. The rate then rises to a high density during an excitatory period where a new spindle is most likely to be generated, at which point the density decays until another spindle is observed.

It is important to note that sleep stage/depth and SO-phase produce densities with fluctuations ranging from ~ 5 to ~12 events/min. This suggests that these factors identify periods in which spindles are more or less probable but are not particularly informative about individual spindle timing. In contrast, the range of the spindle rate for the history model is around three times greater, spanning from a minimum near zero to a maximum around 20 events/min. This suggests that the times of past events predict spindle times and nonspindle times with a much higher certainty than the other factors.

Last, by integrating all information from sleep stage/depth, phase, history, and the interaction between phase and stage (bottom two panels), we can assess how these factors interact to provide a more nuanced instantaneous density. Overall, the history component provides the bulk of structure, with SO phase adding small fluctuations in the density with each slow oscillation, along with other small changes from stage/sleep depth. By integrating information from multiple sources, the density range expands further, varying between 0 to ~30 events/min. These clear periods of both high and low spindle probability suggest that the model can better capture key aspects of spindle temporal dynamics.

Using this integrated approach, we next examine the components of the model to describe each aspect of the influence, their interactions, and their relative contributions to spindle timing.

### 1.3. Spindle Timing Patterns: Past Spindle Activity Influences the Next Spindle.

Temporal patterns of neurophysiological events are crucial in neuroscience as they reflect key aspects of function and underlying mechanisms. Using our framework, we can formally address the degree to which spindle timing exhibits regular temporal patterns. To do so, we incorporate *history dependence* into our models, which can be defined as the influence of past spindle events on the current spindle. In this model, the likelihood of a spindle at each time depends on the time since the previous event. This is a causal model, based only on past information. Moreover, it captures the impact of previous events while fully accounting for the other simultaneously observed factors.

**Fig. 2.**
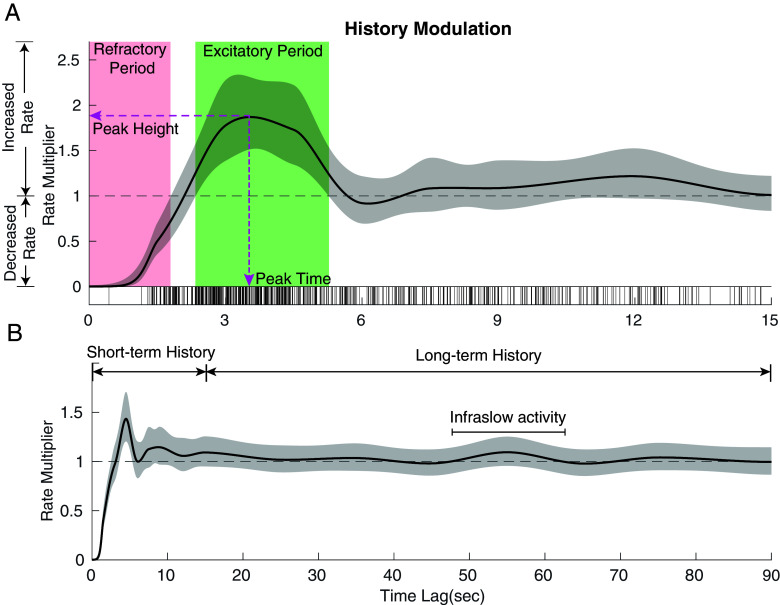
History modulation curve captures spindle timing patterns. (*A*) Using a participant from the MESA dataset, the history modulation plot (solid black curve) shows the multiplicative effect on event rate based on the time since the last event, with 95% CI (shaded in gray). The vertical lines at the bottom are interspindle-intervals. We summarize the history curve by defining the refractory period, excitatory period, peak height, and peak time. (*B*) We extend the model to 90 s to capture spindle long term dependence, including infraslow activity.

We visualize this component of our integrated model using a *history modulation plot* (Fig. 2), which quantifies the multiplicative effect of previous spindle activity on spindle dynamics. The *x* axis shows the time lag since the previous spindle and the *y* axis shows how much more likely a spindle is to occur now if there was a spindle at that lag in the past. This means that values greater than 1 indicate an enhanced spindle rate, values less than 1 indicate a suppressed spindle rate, and values equal to 1 indicate no change. Since the curve is the multiplicative effect on the baseline spindle rate, a value of *m* at time *t* can be thought of as saying, “If we observed a spindle *t* seconds ago, we are *m* times more likely to observe a spindle now.” The baseline spindle rate to which the multiplier is being applied is determined by whatever combination of sleep stage, SO phase, or other factors are present at that time.

The Fig. 2*A* shows a short-term (<15 s) history modulation plot for a participant from the MESA dataset. The history curve (solid black) with a 95% (CI, shaded) are displayed. The curve starts close to 0, rises to a peak at ~3.5 s, and then decays down to 1. When the curve is close to 0, this means we are multiplying the density by a very small number, indicating a refractory period during which few or no events occur. When the curve rises at to a value of ~2 at around 3.5 s, this means that a spindle occurring 3.5 s ago makes it two times more likely to occur now. Significant peaks in the history modulation curve that are above 1 indicate excitatory periods during which spindle density is significantly increased. The curve then falls back down to 1 after ~6 s, which suggests that a spindle occurring between 6 and 15 s ago has no effect on a spindle occurring now, for this participant.

We can also quantify key features of the history structure. Regions of the curve that are significantly above or below 1 can be used to define refractory (red region) or excitatory (green region) periods, respectively. Moreover, we can quantify the height and time lag for the excitatory peak. For this participant, we observe a refractory period of 1.8 s, and an excitatory period of 2.9 s with peak height = 1.9 and peak time = 3.5 s. The details of history feature quantification are provided in *SI Appendix*, Methods.

Spindle activity has also been reported to exhibit clustering patterns on an infraslow (~50 s) scale (4, 16). To account for spindle long-term dependence, we extend our model to 90 s, which includes the infraslow period. In Fig. 2*B*, we observe a small bump at ~55 s, indicative of spindle infraslow activity within the general population. However, the bump does not reach statistical significance, suggesting limited influence on the spindle timing.

### 1.4. Participants with Similar Spindle Density Can Exhibit Different Timing Patterns.

**Fig. 3.**
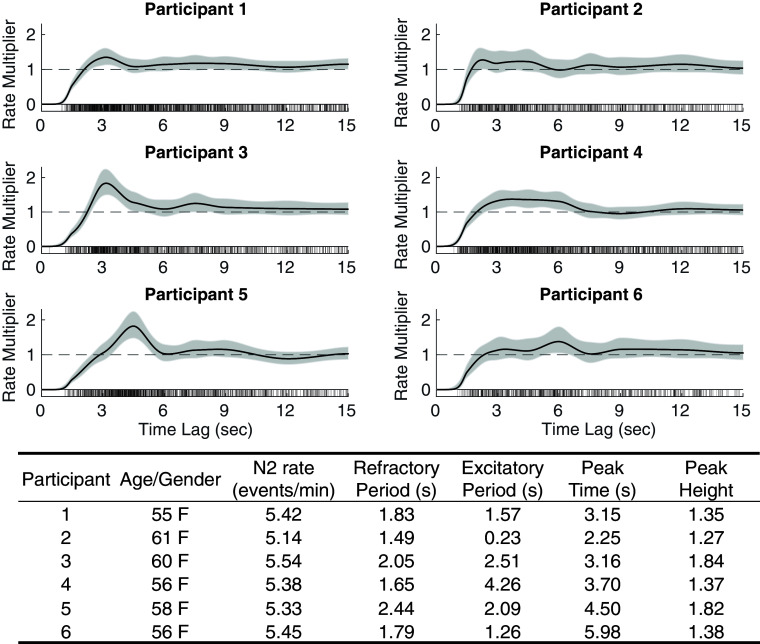
Participants with similar spindle densities can have drastically different history dependence structures. Six female participants are shown with very similar spindle rates (N2 rate: 5.5 ± 0.5 events/min) who possess different history dependence architectures. History dependence curves are shown as solid black lines and 95% CI are shaded in gray, the vertical lines at the bottom are interspindle-intervals. In the *Bottom* panel, the summary table shows age/gender information and history features of each participant, including refractory period, excitatory period, peak time, and peak height.

In Fig. 3, we show six female participants of similar age (age range: 55 to 65 y) from the MESA dataset that have nearly identical spindle density (N2 rate: 5.5 ± 0.5 events/min). Despite these similarities, these participants exhibit temporal patterns with marked structural differences.

All participants share the same general form of history modulation: a refractory period followed by a peak of increased propensity of events, which then decreases to 1. However, the structure of these features varies from subject to subject. For example, the tall peaks exhibited by participants 3 and 5 suggest more periodic trains of events. However, they have vastly different refractory periods and peak times, suggesting differences in periodicity. The broad, low peak exhibited by participant 4 suggests larger variability and randomness in event patterns. Additionally, in examining differs between participants 2 and 4, we see participant 2 has a shorter excitatory period and peak height, suggesting even less predictable events than participant 4. Overall, variation in history dependence structure reflects remarkably heterogeneous temporal patterns, despite similar spindle densities.

### 1.5. Spindle Timing Patterns Are Consistent Night-to-Night but Heterogeneous in Populations.

**Fig. 4.**
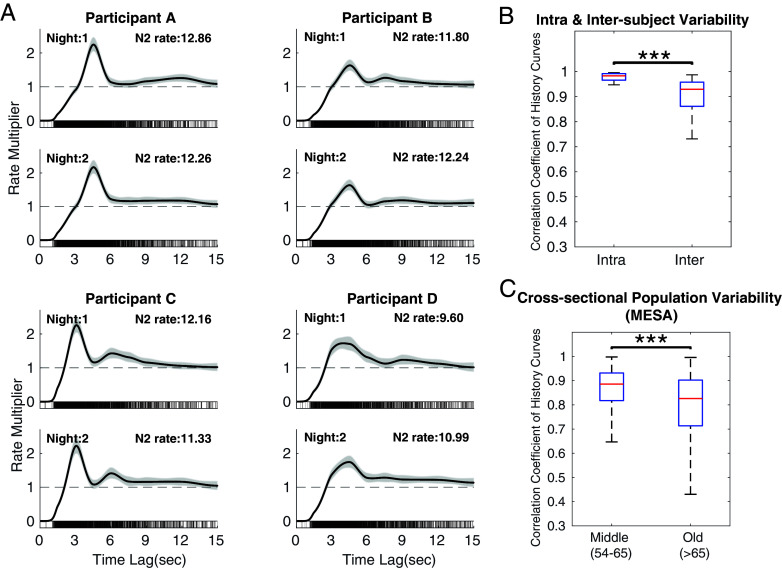
History dependence creates unique “temporal fingerprints” for each participant’s spindle activity, which suggest a basis for characterizing spindle timing phenotypes. (*A*) History curves of four participants across two nights of sleep. (*B*) Boxplots of the correlation coefficients between history curves for the same participant across two experimental nights (intrasubject) and between participants (intersubject) using the first night from the Wamsley dataset (17 participants from two nights). (*C*) Cross-sectional population variability in MESA. Middle-aged group includes 433 participants with age range 54 to 65 y, and the older group has 575 participants with age >65 y. *t* tests are performed to compare group means, where *, **, *** denote *P* values <0.05, <0.01, <0.001, respectively.

Characterizing spindle history dependence structure helps us assess the heterogeneity across participants and consistency within participants across multiple nights. Fig. 4*A* shows four healthy control participants from a younger dataset (N = 17) from Wamsley et al. (22) with recordings from two consecutive nights that have similar N2 spindle density (~11 events/min). Again, we see substantially different levels of variability and periodicity of spindle timing patterns across all 17 subjects, yet night-to-night variability is small within subjects (*SI Appendix*, Fig. S1).

To quantify similarity, we can compute the Pearson’s correlation coefficient between any two history curves. Using the Wamsley dataset, we compute the intrasubject (pairwise correlation between night 1 and night 2 for each participant) and intersubject (pairwise correlation between night 1 curves for all participants). In Fig. 4*B*, intrasubject level shows a mean correlation coefficient of 0.98, which is significantly higher than mean intersubject variability of 0.90 (*t* test), with a much smaller variability in the intrasubject distribution.

We further show the intersubject variability in each age group in the heterogeneous MESA population (1,008 participants, single night), which we separate into middle aged (54 to 65 y) and older (>65 y) groups. In Fig. 4*C*, we observe a significantly lower correlation coefficient and larger variability in the older age group (*t* test). Notably, correlation coefficients in all groups are highly positive in general (mean values > 0.8), indicating that despite vast heterogeneity among populations, most participants share a consistent pattern that commonly involves a refractory period followed by an excitatory period. Overall, history dependence creates an individualized temporal signature of spindle activity and demonstrates strong within-subject stability across nights, which can potentially serve as a quantitative basis for future spindle timing phenotype exploration and clinical biomarker development.

### 1.6. Spindle Temporal Patterns Show Robust Gender and Age Differences in A Large Heterogeneous Population.

**Fig. 5.**
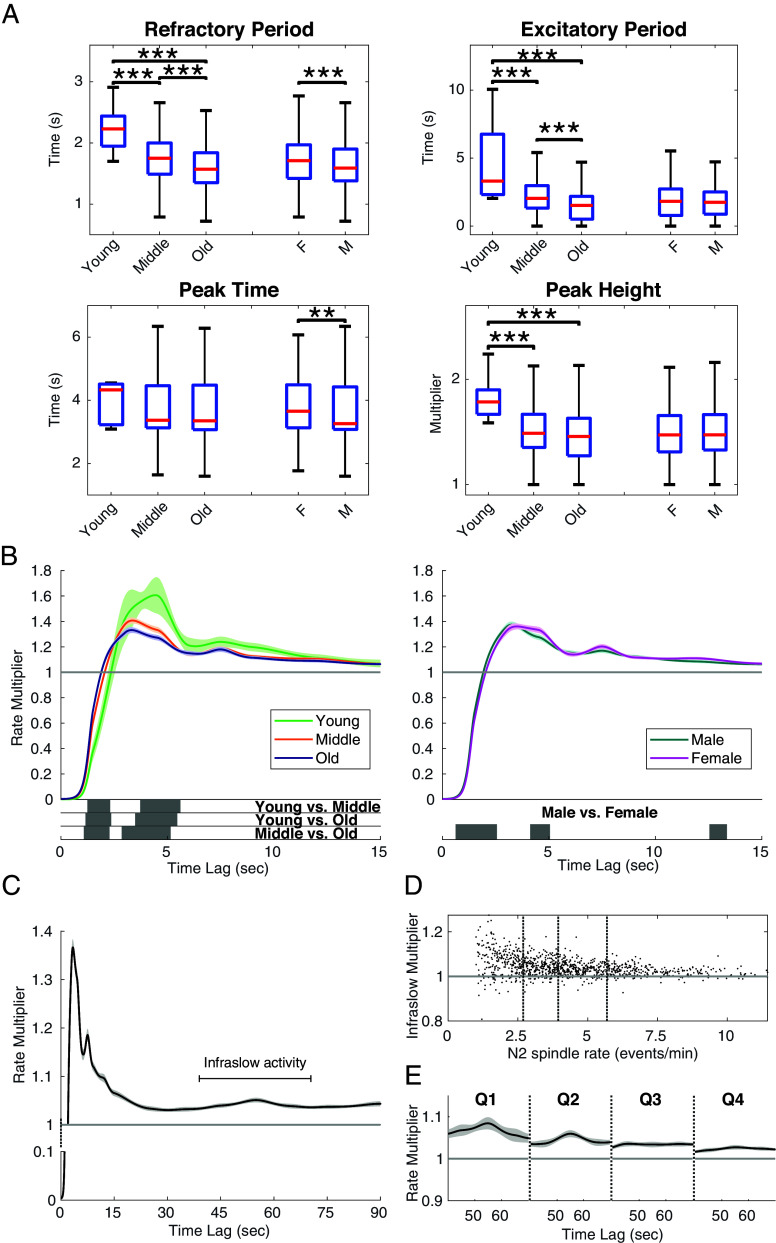
Spindle history dependence shows robust demographic differences and exhibits infraslow activity. (*A*) Comparisons of all summary statistics of history modulation across groups. (*, **, *** denote *P* values <0.05, <0.01, <0.001, respectively for t-tests comparing group means, corrected for multiplicity). (*B*) The mean history modulation curves with 95% confidence bounds for different age and gender groups. The bottom gray regions indicate significant differences using global permutation tests (Significance level: 0.05). (*C*) The mean history curve in the long-term (90-s) across all participants. (*D*) A scatter plot of infraslow multiplier (measured as the normalized area under history curve during infraslow period) vs. N2 spindle rate. (*E*) The average of history curves in each N2 rate quantile group.

Given the ability to quantify the individualized spindle timing patterns, we can characterize the variability in history features over populations. In Fig. 5*A*, we see the refractory period is significantly shorter in the older age groups. One possibility is that this reduction in the refractory period is due to the shorter spindle duration in older people as reported in multiple previous studies (8, 29–32). However, after accounting for spindle duration, we find that the refractory period continues to exhibit a persistent and statistically significant negative correlation with aging (*SI Appendix*, Fig. S2). This suggests that independent of duration, there are significant changes in the intrinsic timing properties of spindles, contributing to a shorter refractory period in older adults.

We also find that older adults exhibit a significantly lower peak height and shorter duration of the excitatory period compared to younger adults. This suggests that spindle patterns are less periodic and more variable in the older people, providing additional evidence of age-related change of sleep spindle dynamics (10, 17).

Distinct differences in history curves are also observed among gender groups, where males exhibit significantly shorter refractory periods and earlier peak times. Moreover, spindle patterns in older female participants generally have similar properties to younger males, which is a common observation in sleep data (8, 33). The correlation between these extracted history properties and other key spindle morphology features is shown in *SI Appendix*, Fig. S3.

In Fig. 5*C*, we observe a broad, low-amplitude bump at around 50 s, providing evidence of infraslow influences on spindle timing. Further analysis reveals that the infraslow multiplier as measured by the area under history curve (34) between 40 to 70 s has a significant negative correlation (ρ= −0.360, *P* < 0.0001) with N2 spindle rate, such that the mean and variance of the modulation increase as spindle rate decreases (Fig. 5*D*). Additionally, no clear infraslow modulation peak is observed at the population level above the median N2 rate (Fig. 5*E*). This indicates that the infraslow rhythmicity is most apparent in those with lower-than-average spindle activity. However, we do not observe significant demographic differences for the infraslow structure (*SI Appendix*, Fig. S4).

It should be noted that a small peak is observed at ~8 s within the population history curves. This secondary peak is likely caused by skipped events, which will create a peak at twice the main peak time. This is primarily due to imperfect event detection in some participants, such as Participants B and C in Fig. 4. Future work can potentially leverage history dependence to improve detection results or employ more agnostic approaches to waveform identification (2, 35).

### 1.7. SO/Spindle Coupling Shows A Continuous Negative Phase Shift with Increasing Sleep Depth.

While modeling spindle history dependence allows us to better characterize temporal patterns, many other factors also impact spindle occurrence. Perhaps the most notable feature thought to influence spindle timing is the SO phase, which is thought to reflect cortical up/down-states. It has been widely reported that fast spindles tend to occur at a preferential phase at the peak of SO (cortical up-state) (10, 12, 27, 36–38), and varies across sleep stage (4, 9).

**Fig. 6.**
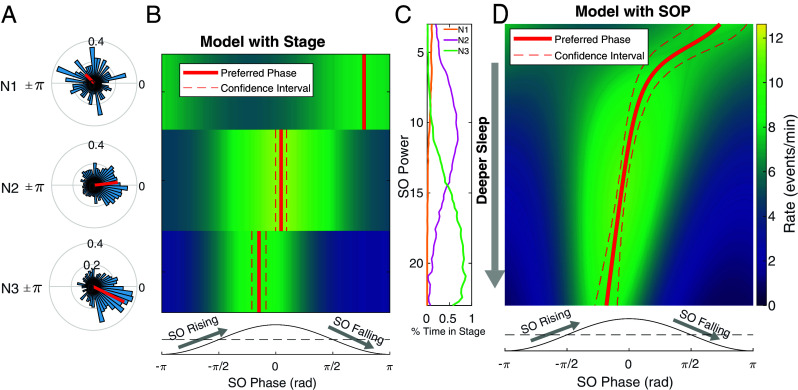
Analysis of spindle/SO coupling as a function of sleep depth for a single participant. (*A*) Polar histograms of spindle phase in stages N1, N2, and N3, with mean phase (red arrow). (*B*) Colormap of the fitted spindle rate as a function of SO phase (*x* axis) and sleep stage (*y* axis). The red solid and dashed lines indicate the estimated preferred phase and its CI from the point process model. The bottom schematic indicates SO rising and falling phase. (*C*) The percentage of time the participant was in scored sleep stages N1, N2, and N3 as a function of the normalized SO power. (*D*) Colormap of the fitted spindle rate as a function of SO phase (*x* axis) and SO power (*y* axis). The red solid and dashed lines indicate the estimated preferred phase and its CI.

We address sleep stage-specific phase coupling by incorporating sleep stage, SO phase, and stage-phase interaction terms into our models. This approach provides us with an estimate of the stage-specific preferred phase with statistical uncertainty and allows us to perform hypothesis tests to determine whether SO/spindle coupling depends on sleep stage. In Fig. 6*B*, the solid red lines show the preferred phase in each sleep stage and the dashed red lines show the 95% CI for a single participant. We observe that the preferred phase shifts from the peak of the SO (~0 phase) to the rising phase of SO (~ −π/8 phase) as this participant moves from stage N2 to N3. Sleep stage has a statistically significant influence on SO/spindle coupling ($\chi^{2}$ test, *P* < 0.0001) for this participant. Note that since there are very few events detected in N1 stage (Fig. 6*A*) this creates large uncertainty in phase preference (CI spans −π to π), suggesting that N1 does not have a statistically relevant preferred phase.

As sleep is a continuous, dynamic process, the natural extension is to examine how SO/spindle coupling is influenced by sleep depth when considered as a continuum. Thus, we replace the discrete stages in the previous model with SO power. For the same participant, we see the preferred phase shows a continuous gradient from the SO peak to the SO rising phase as the participant falls deeper into sleep (Fig. 6*D*). This aligns with the result from the model with sleep stage but provides a more precise characterization of the within- and across-stage coupling dynamics, revealing the continuous evolution of SO/spindle coupling with depth of sleep.

### 1.8. SO/Spindle Coupling Shows A Negative Phase Shift across All Sleep Depths in Aging.

**Fig. 7.**
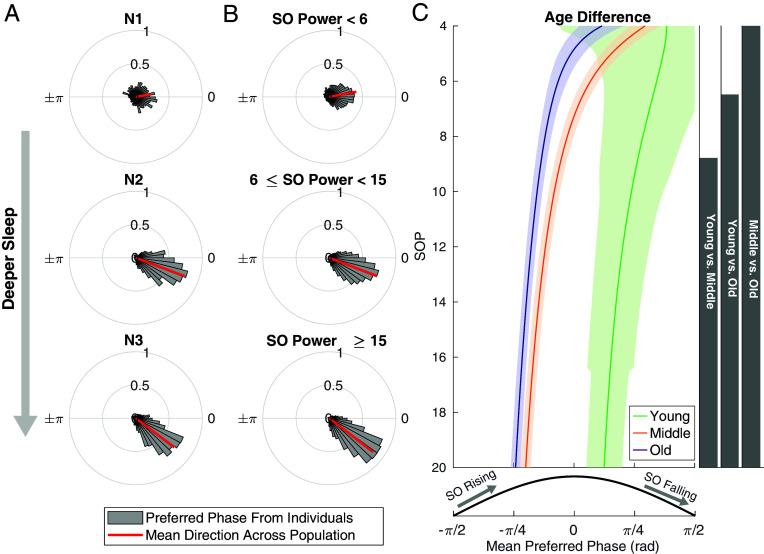
Population trends reveal shifts in preferential phase over sleep depth. Sleep spindles tend to occur at the up-state of SO (peak of slow oscillation, ~0 phase of cosine curve). As participants in this population move to the deeper sleep, their preferential phase shifts toward the down-state (trough) of the SO. The results are shown for both the discrete (*A*) and continuous (*B*) sleep depth metrics. Gray polar histograms show the distributions of preferred phase across the population, with the red line showing the mean direction of the polarhistogram. (*C*) Mean phase shift curves from the model with SOP-phase interactions are shown across three age groups (Young: 26 to 45 y from Wamsley, green; Middle: 54 to 65 y from MESA, orange; Old: >65 y from MESA, blue), with significance regions determined by global permutation tests for all combinations (Significance level: 0.05).

We examined the spindle phase dependency across the MESA cohort. $\chi^{2}$ tests show 839 out of 1,008 participants (83.23%) had significant dependence between spindles and SO phase. We further assessed sleep depth-dependent coupling across these 839 participants and compared the results over middle (54 to 65 y) and old (>65 y) age groups in MESA cohort, as well as in a much younger Wamsley group (age range: 26 to 45 y). Fig. 7 *A* and *B* show the distribution of the preferred phase (gray polar histograms) and the mean preferred phase (red line) across each sleep stage and SOP category. As people move to deeper sleep, we see a consistent and statistically significant shift (Watson-Williams tests, *P* < 0.0001) of the preferential phase from the peak of the SO to the rising slope of the SO.

Though evidence of stage-dependent phase coupling has previously been reported in studies from Cox et al. (9), due to the small sample size (11 participants were included in N3, and 14 participants in N2) and their method for computing preferred phase, the authors did not have sufficient statistical power to identify a significant difference in fast spindle/SO coupling between N2 and N3. Our results show the same direction of phase shift from N2 to N3, but also provide a statistically powerful way to detect small shift patterns that would not be identifiable through simple summary statistics.

We also characterize the phase coupling as a function of SOP in different age groups. Fig. 7*C* shows the mean phase coupling curve across middle and old age groups in the MESA dataset and the younger group from Wamsley et al. (22). We observe a global negative phase shift across all depths of sleep as a function of age, which is critical because it demonstrates that this shift is not solely due to the age-dependent differences in sleep depth.

To further test whether the phase shift is significant within each age group, we break down Fig. 7 *A* and *B* into young, middle, and old groups. In *SI Appendix*, Fig. S5, we observe the significant negative phase shift from N2 to N3 in the young dataset (Watson-Williams test, *P* = 0.0082), as well as in the middle and old groups (Watson-Williams tests, *P* < 0.0001). Thus, the negative phase shift with increasing sleep depth is a global property across all age groups.

### 1.9. Short-Term History Is The Main Contributor to Spindle Timing Dynamics and Pervasive across Participants.

Having discussed how each of these factors can modulate spindle activity, our modeling framework also allows us to quantitatively compare the relative contributions of these factors through deviance analysis, which is the point process equivalent of an analysis of model variance in linear regression. By comparing nested models for all MESA participants, we compute the percentage of the deviance in the full model explained by adding each subsequent factor (see *SI Appendix*, Methods for details). We find that among all factors, the short-term history contributes the most at ~70% (population mean: 72.11%, 95% CI [71.15%, 73.07%]) of the fractional deviance explained, compared to sleep depth (sleep stage: 16.58% [15.90%, 17.47%]; SOP: 18.33% [17.23%,19.42%]) and slow oscillation phase (13.60% [12.88%,14.31%]). Adding long term history provided an approximately 5% improvement (77.91% [77.12%, 78.69%]) over short-term history.

We also looked at the population percentage of each model that passed a goodness-of-fit analysis (Kolmogorov–Smirnov test), demonstrating that they were able to capture the observed pattern of spindle events without significant evidence of model misfit. Single factor models of sleep stage, SOP, and phase models passed in 14.29%, 19.21%, and 16.07% of the population, respectively. Using history alone, however, passed in 72.02% of the population. Finally, the model of SOP, phase, and history passed in 98.71% of the population, suggesting the additive importance of multiple factors and the primacy of short-term history.

In addition, we evaluate the pervasiveness of spindle short-term history dependence in the MESA cohort. $\chi^{2}$ tests reveal that 991 out of 1,008 participants (98.31%) exhibit a statistically significant history effect, suggesting that short-term history dependence is a near-universal feature of spindle activity. Adding long-term history, about half (51.59%) of the entire population has statistically significant model improvement ($\chi^{2}$ tests).

### 1.10. Infraslow Activity Has Limited Impact on Spindle Timing.

Given the ~5% increase of deviance explained by adding from long-term history, it is of interest to see the specific impact of the infraslow period. To do so, we fit additional models with history out to infraslow period in addition to the full 90s model (see *SI Appendix*, Methods for details). From this analysis, we conclude that the infraslow period contributes less than 1% (0.74% [0.67% 0.81%]) to the decrease in deviance. This suggests that infraslow clustering, while significantly detectable over a night, has a very limited impact on the timing of any individual spindle.

### 1.11. History and SO Phase Have Independent Influences on Spindle Dynamics.

Given the large amount of information gained by the inclusion of spindle history dependence, one mechanistically critical question arises: Do history dependence and SO phase carry redundant information about spindle timing? It could be that history is incorporating the inherent timing of the slow oscillation. We directly address this question by computing the synergy index, a common measure in neural modeling derived from information theory (39–42), where value 0 indicates entirely independent information provided by two components (See *SI Appendix*, Methods for synergy index). Computed across the population (*SI Appendix*, Fig. S6), the distribution of the synergy index is tightly concentrated around 0 (mean: −0.0023 ± 0.0024), which suggests that history and phase provide mutually independent information in modulating spindle activity. As an alternative approach, a mean correlation matrix of fitted parameters across the MESA cohort from the multifactor model with stage, phase, and history is shown in *SI Appendix*, Fig. S7. Again, we see that SO phase and history components have almost no correlation with each other. Overall, these results strongly suggest that short-term history is providing timing information independent of SO phase.

## 2. Discussion

Sleep spindle activity is a dynamic process in which the rate and timing of events are simultaneously governed by multiple factors and their interactions, the intricacies of which are not fully understood. In this work, we developed a quantitative approach to characterize the factors contributing to spindle timing. We model the instantaneous spindle density as a function of multiple simultaneously observed influencing factors, including sleep stage, past spindle history, slow oscillation activity, and their interactions. Results reveal fingerprint-like timing patterns, characterized by a refractory period followed by a period of increased spindle activity, which are highly individualized yet consistent night-to-night, with increased variability with age. Strikingly, short-term (<15 s) temporal patterns of past spindle history are the main determinant of spindle timing, surpassing the contribution of factors known to affect overall spindle density such as cortical up/down-state (slow oscillation phase), sleep stage, and long-term history (15 to 90 s). Short-term history and slow oscillation phase exert independent effects on spindle timing. Our results suggest informative features of spindle patterning indicative of natural heterogeneity and potentially informative about pathophysiological processes.

### 2.1. Spindle Activity Is History Dependent.

Our results suggest that spindle production is history dependent, such that the timing of a spindle is influenced by the timing of previous events. This effect is pervasive, with nearly all participants sharing a similar general history modulation structure starting with a refractory period in which events do not occur, followed by an excitatory period, in which there is an increased likelihood of events. This can lead to a sequence of high-density spindles: As multiple events arrive sequentially, the history of multiple past events can interact, with excitatory and refractory periods combining constructively or destructively depending on spindle timing. The structure of these history-dependent factors creates patterns in spindle timing, variation in which creates differences between individuals. Like other features of spindle-like transient oscillations (2, 9), the history-dependent structure exhibits strong night-to-night consistency for individuals, with intersubject heterogeneity, even for participants with similar N2 spindle density. This suggests that the history curve, as a quantitative fingerprint, could serve as a diagnostic tool which may be related to functions of sleep spindles such as synaptic plasticity, sleep-dependent memory consolidation, and sleep stability and be informative in the context of diseases where spindle abnormalities are present (4).

We observe robust differences in history-dependent structure across gender and age groups. In particular, we show that females are more robust to alterations in history with the aging process, which may align with previous findings showing males experience worse sleep disruption and NREM sleep impairment than females (8, 33, 43). We characterized spindle refractoriness in a large human dataset. We show that the spindle refractory period shortens with aging independently of age-related reductions in spindle duration. The reduction of the excitatory period and peak height in older groups suggests a loss of spindle rhythmicity and an increase of randomness in the elderly. This corroborates previous findings (17) showing decreased spindle clustering with aging, but provides a complete and unbiased characterization of spindle temporal structure without relying on hard thresholds to define the temporal clustering level. Overall, these findings show the potential for this model framework to address specific epidemiological questions and mechanistic hypotheses in future applications.

### 2.2. Interacting Windows of Opportunity.

Our analysis and modeling approach provide several results that lead us to reevaluate the existing perspective on spindle production—in particular, the role of the cortical up-state in driving spindle timing may not be as essential as previously thought. This is illustrated clearly in Figs. 1 and 6*A*, which highlights that while more spindles occur in the up-state of SO, many other spindles also appear at other phases or even in the SO down-state. Moreover, there will be thousands of cortical up-states during a night at which no spindles will occur. Overall, this suggests that spindle production is a complex and multifaced process, involving multiple factors at different time scales, each of which contributes to providing enhancement or suppression of spindle likelihood at any given point in time. Thus, it is the combination of many factors that provide various windows of opportunity for spindle activity to occur. The dominance of history dependence from deviance analysis implies that intrinsic factors of spindle production such as refractory/excitatory periods may override extrinsic triggering factors such as SO-phase.

### 2.3. The Assumption of Spindle/Slow Wave Co-occurrence Imposes Bias on Mechanistic Interpretations.

Several possibilities could contribute to this alternative viewpoint on spindle production. In particular, the pervasive practice of requiring the co-occurrence of discrete spindles and slow-waves in coupling analyses for both EEG and intracranial studies could deeply bias hypotheses (A schematic plot shown in *SI Appendix*, Fig. S8). Methodologically, this approach consists of independently detecting spindles and slow waves, then restricting the analysis to only those detected spindle events that sufficiently temporally overlap with detected slow wave events. These waveform detection algorithms have been shown to be highly variable in their results (1, 12, 28, 44) with ad hoc parameter settings that can create rarity assumptions that underselect events with low amplitude but identical morphology (1). For this reason, studies often only examine SO/spindle phase coupling in deep NREM sleep (N3 to N4), so that they may find enough slow waves that pass the detection threshold. Consequently, low co-occurrence rates have been explicitly noted during N2 (36). However, this is suboptimal since fast spindle activity is definitionally maximal in N2 sleep (4), so the majority of spindles throughout the night are being ignored. Selection bias is further exacerbated by the common practice of further restricting the analysis to only the detected spindles of the largest amplitude (e.g., top 25%).

Beyond these methodological issues, forced co-occurrence raises theoretical questions. By removing any slow waves and spindles that do not co-occur, this approach imposes the assumption that spindles without slow waves are not functionally important. Given a scenario in which SO-phase intermittently or weakly contributes to spindle production, as suggested by the population model analysis results, co-occurrence would definitionally induce a stronger importance of phase coupling. Thus, the choice of many studies to focus on spindle/slow-wave coupling requires further study and justification.

Crucially, the co-occurrence assumption directly impacts the ability to observe history dependence by drastically reducing the chance of observing runs of consecutive spindles over consecutive slow waves. Without these runs, it is impossible to see short-term history processes like refractory periods and excitatory periods, as co-occurring spindles will rarely be close enough temporally to interact with previous events. Consequently, thus far, the timing of spindles and their phase coupling have had to be examined separately, as co-occurrence eliminates any dependence on previous events or history. In using our approach, we are now able to measure the relative influences of these processes on spindle timing.

### 2.4. Dynamic Spindle/SO Coupling over Sleep Depth.

Our study provides a conclusive demonstration of significant spindle/SO coupling shifts dynamically in a continuum with sleep depth (sleep stage & SOP) in a large dataset. This agrees with existing studies reporting phase coupling in N2 and N3 (4, 10, 36, 37, 45), as well as those suggesting differences but lacking the statistical power to show the difference (9). Moreover, we expand upon previous studies (10, 36, 37) showing a fixed phase shift toward the rising edge of the SO with age, by showing that phase coupling dynamics as a whole is largely preserved with age, and that the whole continuum is globally shifted earlier.

Note that the large CI observed in the young group in Fig. 7*C* is due to the small dataset (N = 17) and very few spindles occurring during light sleep. Another caveat of phase comparison is that part of the analysis involves datasets from different sleep studies. Cross-study comparisons of phase can introduce significant bias due to differences in filters and systems. Nonetheless, our results demonstrate significant negative phase shifts within each age group (*SI Appendix*, Fig. S5), with the direction of the shift aligning with previous literature (10). Future work will comprehensively assess this across larger datasets spanning the entire age spectrum.

### 2.5. Implications for Spindle Generation Mechanisms.

Studies in experimental animals provide some clues about the physiological mechanisms underpinning our observations. Sleep spindles recorded from cortical sites are generated through the interplay of neuronal activity in GABAergic thalamic reticular nucleus (TRN) neurons, especially the major population containing the calcium binding protein parvalbumin (46–48) and glutamatergic thalamocortical (TC) neurons which in addition to projecting to the cortex also send collateral projections that feedback and excite TRN neurons (4, 49). During wakefulness, both TRN and TC neurons are relatively depolarized due to the excitatory action of ascending neuromodulatory inputs. When these inputs are withdrawn during NREM sleep, the ensuing hyperpolarization brings TRN and TC neurons into the appropriate voltage range where low-threshold calcium channels (Cav3.3 and Cav3.2 in TRN neurons, Cav3.1. in TC neurons) can become active. Activation of low-threshold calcium channels leads to burst discharge of TRN neurons and a compound inhibitory postsynaptic potential (IPSP) in TC neurons which hyperpolarizes them and removes inactivation of low-threshold calcium channels. Once the IPSP decays, these channels are activated, leading to a burst discharge of TC neurons, resulting in activation of cortical neurons and activation of TRN neurons, restarting the cycle of the spindle oscillation. Various biophysical and synaptic connectivity properties of TRN and TC neurons affect the waxing and waning of spindles, their amplitude, frequency, and duration (4, 50) and likely contribute to the individual differences in refractory period, peak time, and peak height we observe in the short-term history curves here.

In contrast to the mechanisms underlying the generation of spindles themselves, less research has focused on the mechanisms regulating temporal spindle patterns. Over long timescales (hours), spindle occurrence is regulated by homeostatic and circadian mechanisms (4, 51). Neuromodulatory inputs from serotonergic raphe neurons and cholinergic basal forebrain and brainstem neurons may also play a role in spindle occurrence, related to their control of the depth of sleep and transitions between sleep stages (49). Recent studies in mice have also described an infraslow (~50 s) cycle regulating the likelihood of spindle occurrence which is controlled by the activity of locus coeruleus noradrenaline neurons (14, 15).

While we see evidence of spindle rhythmicity at the infraslow level, many questions remain about spindle infraslow activity. For example, the mean history curve does not return to 1 even after extending the model to 90 s, indicating the presence of even slower dynamics beyond this time frame. Moreover, the subjects with the highest infraslow influences in history curves often had EEG with poor signal quality and low spindle density. This is consistent with our finding of inverse relationship between infraslow magnitude and N2 rate. Overall, this suggests that infraslow activity either has a weak effect on spindle timing masked by sufficient spindle activity, or it is the product of external factors or noise. Future analyses should take careful consideration to understand the impact of nonneurophysiological factors in infraslow clustering and develop methods to quantify long-term history dependence at the individual level.

In vitro studies of in TRN and TC neurons suggest possible ionic mechanisms underlying the short-term history dependence of spindles, especially the refractory period. In TRN neurons, a study in ferret brain slices in vitro described a slow afterhyperpolarization of TRN neurons following repetitive bursting caused by sodium and calcium-dependent potassium currents (52). In TC neurons, in vitro studies in rats and cats (53, 54) have described a pronounced afterdepolarization following repetitive burst discharge due to prolonged activation of hyperpolarization and cyclic nucleotide gated cation (HCN) channels which generate the so-called H-current. This afterdepolarization will prevent further burst discharge of TC neurons, which is required for spindle generation by increasing inactivation of low-threshold calcium channels and taking the membrane potential of TC neurons out of the range in which they can be activated. The afterdepolarization is caused by increased intracellular calcium due to entry through low-threshold (Cav3.1) calcium channels, leading to calcium-activated production of cyclic AMP, which activates HCN channels (55–58). Large scale computational models of thalamocortical activity underlying sleep spindles also point to calcium modulation of HCN channels as an important controller of spindle refractoriness (59). Following the refractory period, we describe a variable period of enhanced spindle likelihood. The mechanisms responsible for the rebound excitation are less clear but may involve deinactivation of low-threshold calcium currents that occur during the refractory period, short-term synaptic plasticity of TRN-TC, TC-TRN, or cortical inputs to TRN, as well as alterations in cortical input to TRN. Factors which affect the synchronization of TRN and TC discharge may also affect the amplitude and form of the rebound excitation. Computational models will be helpful in understanding the relative importance of these factors (60–62).

The factors responsible for interindividual differences in the temporal history of spindles and the effect of biological sex, age, and disease remain to be uncovered, but as previously described are likely due to differences in the biophysical properties of TRN and TC neurons, their intracellular sodium and calcium dynamics, their synaptic connectivity, kinetics, and plasticity, with each other and with the cortex (4, 63, 64), as well as interactions with ascending neuromodulatory and GABAergic inputs to the TRN and TC neurons (49, 65, 66). Our study, which provides a robust unbiased framework to describe the temporal history of spindles, sets the stage for such mechanistic/genetic studies in humans and potentially also in animals, which can uncover the basis for inter- and intraindividual differences and the effect of learning on these dynamics.

### 2.6. Analysis Limitations and Extensions.

In this study, we focused on fast spindle analyses from single-channel EEG recordings. However, previous studies have shown topological differences in spindle/SO coupling (31, 67), as well as distinct types of SOs coupled with fast and slow spindles (4, 68–70). Preliminary results suggest differences in preferred coupling phase for central fast spindles contingent on the electrode from which the SO is computed (*SI Appendix*, Fig. S9), but a comprehensive study must be performed to determine the degree to which the low predictive power of phase is confounded by the aggregation of topologically distinct waveforms within a single channel. Future work will extend this framework to large high-density EEG datasets to further explore spatiotemporal differences in spindle dynamics.

Researchers have also reported a three-way coupling between spindles, SOs, and hippocampal ripples (11–13, 28). While we are not able to detect ripples from normal sleep EEG, this framework can be applied to intracranial data to better understand the mechanisms of such higher-order associations. Additionally, other sleep events like K-complexes, periodic limb movements of sleep, and arousals may also be linked to spindle dynamics. Future work will expand the model components to explore these associations.

In this study, we assumed that the history curve remains consistent across different sleep stages, given definitionally low spindle rates outside of N2. Preliminary analysis (*SI Appendix*, Fig. S10) incorporating stage–history interaction shows that individuals with low N3 spindle rates show extremely large uncertainty in the history curve estimates. To address this, we excluded 182 out of 1,008 (18.06%) subjects with <1 event/min in N3. In those with sufficient N3 rates, we observe reduced history dependence in N3 sleep, with 24.46% (202/826) of the MESA population showing statistically significant stage-dependent history effects ($\chi^{2}$ test). Future analysis will incorporate SOP–history interaction term to capture changing history patterns of spindles with continuous sleep depth.

### 2.7. Conclusion.

In this study, we have developed a rigorous statistical framework for the analysis of the relative contributions of numerous factors involved in spindle production. We have shown that short-term timing patterns provide the most significant predictive information in spindle timing, compared to other well-known factors, including cortical up/down-state, sleep depth, and infraslow activity. The short-term timing patterns are fingerprint-like, exhibiting remarkable individualization across participants yet demonstrating strong consistency within participants across nights, with distinct differences across age and gender groups. Mechanistically, our study provides insights into spindle production, suggesting future experiments further elucidating the role of spindle timing patterns in health and disease. Methodologically, we offer a statistically principled framework to tackle fundamental spindle questions in a rigorous way, which could facilitate studies of sleep-dependent memory consolidation, characterize spindle abnormalities in neuropsychiatric disorders, and contribute to the development of sleep biomarkers.

## 3. Methods

### 3.1. Data Description.

We analyzed data from the MESA (23, 24), obtained from the National Sleep Research Resource (www.sleepdata.org). EEG data were from electrode C4-M1, sampled at 256 Hz. We limited our analysis to 1,008 participants (male/female: 523/485, age mean: 68.74 ± 8.95) that had high EEG quality (EEG signal good >= 95% of sleep time) and a spindle rate of at least 1 event/min in N2 or N3 stage. We also analyzed data from a previously published study by Wamsley et al. (22) that included two-night sleep EEG recordings from 17 healthy participants. EEG data were from the C3 and F3 electrodes (linked mastoids), sampled at 100 Hz. See *SI Appendix*, Methods for details on data processing and event detection.

### 3.2. Approach Overview: Spindles as Point Processes.

Point processes are mathematical models that describe discrete events that occur in space or time, which have been successfully applied in numerous fields (21, 71–73), but have not yet been used to explicitly model sleep spindle timing patterns. A general point process can be defined in terms of a *conditional intensity function* (20, 21), $\lambda \left(t|H_{t}\right)$, which describes an “instantaneous spindle density” at time t, given $H_{t}$, the history of past events up to, but not including time t. In this case, our conditional intensity function will define the moment-to-moment spindle rate, given all the information up to the current time point. We express the conditional intensity function in terms of covariates that influence spindle events, including sleep stage, continuous sleep depth metrics (e.g., slow oscillation power), slow oscillation phase, past spindle events, as well as the interactions between these factors. In doing so, we develop a formal statistical framework to determine quantitatively which factors affect the instantaneous probability of a spindle occurring, the relative contribution of each factor, and how factors interact with others, providing a basis for the identification and analysis of spindle biomarkers. To implement these models, we employ point process-GLMs, a well-studied class of point process models with numerous properties that make them useful for the analysis of spindle event data, including computational efficiency and model interpretability (20, 21, 74). See *SI Appendix*, Methods for details. In general, we define the conditional intensity $\lambda \left(t|H_{t}\right)$ as a function of possible covariates that influence spindle events:

$$\lambda \left(t|H_{t}\right)=f(\text{sleep depth, slow oscillation features, spindle history,...}),$$

In this study, we primarily employ the following mathematical form, which incorporates sleep stage, SO phase, stage-phase interaction, and history:

$$\begin{array}{ll} \log\lambda \left(t|H_{t}\right) & =\overset{\text{sleep stage}}{\overset{︷}{\sum_{s\in S}\beta_{s}I_{s}\left(t\right)}}+\overset{\text{SO phase}}{\overset{︷}{\beta_{1}\cos\left(\phi_{t}\right)+\beta_{2}\sin\left(\phi_{t}\right)}}\\ \; & +\overset{\text{stage-phase interaction}}{\overset{︷}{\sum_{s\in S}I_{s}\left(t\right)\left[\beta_{s,1}\cos\left(\phi_{t}\right)+\beta_{s,2}\sin\left(\phi_{t}\right)\right]}}\\ \; & +\overset{\text{spindle history}}{\overset{︷}{\sum_{k=1}^{K}h_{k}g_{k}\left(H_{t}\right)}} \end{array}$$

In the sleep stage term, the indicator function $I_{s}(t)$ expresses whether a participant is in the corresponding sleep stage $s\in S:\left\{N1,N2,N3,REM,Wake\right\}$ at time t, with parameter $\beta_{s}$. Specifically, $I_{s}\left(t\right)=1\;\mathrm{if}\;\mathrm{stage}=s\;\mathrm{at}\;t$, and 0 otherwise. We can also choose to replace sleep stage with a model of SO power, which we model as quadratic, $\alpha_{1}SOP_{t}+\alpha_{2}SOP_{t}^{2}$. The SO phase term, $\beta_{1}\cos\left(\phi_{t}\right)+\beta_{2}\sin\left(\phi_{t}\right)$, models the density as a cosine tuned function of SO phase, $\phi_{t}.$ This model is mathematically equivalent to $M\cos\left(\phi_{t}-\phi_{\mathrm{pref}}\right)$, with a coupling magnitude $M=\sqrt{{\beta_{1}}^{2}+{\beta_{2}}^{2}}$, and a preferred phase $\phi_{\mathrm{pref}}=\text{atan2}(\beta_{2},\beta_{1})$. The third term models the interaction between sleep stage and phase, in which terms 1 and 2 are combined. The history term fits a cardinal spline to the spindle history, $H_{t}$. K is the number of basis functions and $h_{k}$ are corresponding parameters that determine the shape of history modulation plot. Here, we used cardinal spline basis functions for $g_{k}\left(H_{t}\right)$. See details in *SI Appendix*, Methods.

## Supplementary Material

Appendix 01 (PDF)

## Data Availability

Data and code availability are detailed in the *SI Appendix*. Additionally, two-night data from the study by Wamsley et al. (22) were obtained with permission from the study authors and may be available upon request.
