# Supplementary material for: Individualized temporal patterns drive human sleep spindle timing
Source: Proc Natl Acad Sci U S A. 2025 Jan 7;122(2):e2405276121. doi: 10.1073/pnas.2405276121 (PMC11745340; doi:10.1073/pnas.2405276121)
Supplement: Supplementary file 1 — Appendix 01 (PDF) [file pnas.2405276121.sapp.pdf]

Supporting Information for

Individualized temporal patterns drive human sleep spindle timing

Authors:

Shuqiang Chen[a], Mingjian He[b,c], Ritchie E. Brown[d], Uri T. Eden[e], and Michael J. Prerau[f,g,1]

[a]Graduate Program for Neuroscience, Boston University, Boston, MA 02215
[b]Department of Anesthesia, Critical Care and Pain Medicine, Massachusetts General Hospital, Boston, MA 02114
[c]Harvard-MIT Health Sciences and Technology, Massachusetts Institute of Technology, Cambridge, MA 02139
[d]Department of Psychiatry, Veterans Affairs Boston Healthcare System and Harvard Medical School, Boston, MA 02132
[e]Department of Mathematics and Statistics, Boston University, Boston, MA 02215
[f]Division of Sleep and Circadian Disorders, Brigham and Women's Hospital, Boston, MA 02115
[g]Department of Medicine, Harvard Medical School, Boston, MA 02115

[1]Corresponding author: Michael J. Prerau. Email: mprerau@bwh.harvard.edu.

This file includes:

- **SI Methods**
- **SI Figures S1 to S11**
- **SI References**

- **SI Methods**

Developing a point process framework for spindle events

To establish a point process model, we first define the counting process for spindle events, denoted as $N(t)$, which counts the total number of spindles that occur up to and including time t, where $t \in (0, TST]$ and TST corresponds to the total sleep time (excluding all wake periods during sleep). The spindle density is defined as $N(TST)/TST$.

The conditional intensity function(1) is a measure of the instantaneous probability of a spindle event at each time,

$$\lambda(t|H_t) = \lim_{\Delta \to 0} \frac{P[N(t+\Delta) - N(t) = 1|H_t]}{\Delta}$$

where $\lambda(t|H_t)$ is the conditional intensity at time t, which depends on H_t, the past history of events up to, but not including time t.

For a specific set of times $s_1 < s_2 < \cdots < s_n$ over the interval $[0, T]$, the likelihood of observing spindles at those times is(1):

$$L = \prod_{i=1}^{n} \left[\lambda\left(s_i \middle| H_{s_i}\right)\right] e^{-\int_0^T \lambda(t|H_t)dt}$$

Therefore, the conditional intensity defines the probability distribution of observing a spindle event at each instant, which can be used to compute the likelihood of any pattern of events over the course of the night.

Model identification

Defining a point process model of spindle events involves expressing the conditional intensity, $\lambda(t|H_t)$, as a function of the variables that influence these events. One well-studied class of point process models fits into the broader statistical framework of generalized linear models (GLMs)(1–3). Point process GLMs typically express the log of conditional intensity as a sum of a set of functions of the factors that influence spindles, each multiplied by a model parameter to be estimated:

$$\log\big(\lambda(t|H_t)\big) = \sum_{j=1}^{p} \beta_j g_j(x_t, H_t),$$

where p is number of model parameters, β_j is the jth model parameter, and $g_j(x_t, H_t)$ are a set of functions of the variables, x_t, that influence the intensity of events and H_t, that determine how past history influences the intensity of events.

The complete list of all model forms used in this paper is provided in table 1, where we start by a number of possible single predictors including sleep stage, spindle past history, SO phase, and SO power. We then build up a set of models by combining multiple predictors and including their interactions to create more detailed models.

As described in the Methods 3.2, we primarily use the model form with sleep stage, SO phase, stage-phase interaction, and history throughout the paper. And we replace the stage with SO power when characterizing phase coupling shift with sleep depth as a continuum (Figure 6d, Figure 7b&c). In an additional analysis, we also incorporate a stage-history interaction term to test stage-dependent history effect (SI Fig. S10).

Table 1: Spindle dynamics model list

Component	Model Form $\log(\lambda(t\|H_t))$ =	Notation
null model	$\sum_{s\in Q}\beta_s I_s(t)$	• Q: {NREM, Wake or REM}<br>• $I_s(t)=1$ if stage $= s$ at t, and 0 otherwise<br>• Parameters: $\beta_s,\ s\in Q$
stage	$\sum_{s\in S}\beta_s I_s(t)$	• S: {N1, N2, N3, REM, Wake}<br>• $I_s(t)=1$ if stage $= s$ at t, and 0 otherwise<br>• Parameters: $\beta_s,\ s\in S$
SOP	$\sum_{s\in Q}\beta_s I_s(t)+\alpha_1\mathrm{SOP}(t)+\alpha_2\mathrm{SOP}(t)^2$	• $\mathrm{SOP}(t)$: SO power at time t<br>• Parameters: $\alpha_1,\alpha_2,\beta_s,\ s\in Q$
phase	$\sum_{s\in Q}\beta_s I_s(t)+\beta_1\cos(\phi_t)+\beta_2\sin(\phi_t)$	• ϕ_t: SO phase at time t<br>• Parameters: $\beta_1,\beta_2,\beta_s,\ s\in Q$
history	$\sum_{s\in Q}\beta_s I_s(t)+\sum_{k=1}^{K}h_k g_k(H_t)$	• g_k: Spline basis function<br>• H_t: Spindle history<br>• K: Number of basis function<br>• Parameters: $\beta_s,\ s\in Q; h_k, k=1,\dots,K$
phase, history	$\sum_{s\in Q}\beta_s I_s(t)+\beta_1\cos(\phi_t)+\beta_2\sin(\phi_t)+\sum_{k=1}^{K}h_k g_k(H_t)$	Parameters: $\beta_1,\beta_2,\beta_s,\ s\in Q; h_k, k=1,\dots,K$
stage, phase, history	$\sum_{s\in S}\beta_s I_s(t)+\beta_1\cos(\phi_t)+\beta_2\sin(\phi_t)+\sum_{k=1}^{K}h_k g_k(H_t)$	Parameters: $\beta_1,\beta_2,\beta_s,\ s\in S; h_k, k=1,\dots,K$
SOP, phase, history	$\sum_{s\in Q}\beta_s I_s(t)+\alpha_1\mathrm{SOP}(t)+\alpha_2\mathrm{SOP}(t)^2+\beta_1\cos(\phi_t)+\beta_2\sin(\phi_t)+\sum_{k=1}^{K}h_k g_k(H_t)$	Parameters: $\alpha_1,\alpha_2,\beta_1,\beta_2,\beta_s,\ s\in Q;$<br>$h_k, k=1,\dots,K$
stage, phase, stage-phase interaction, history	$\sum_{s\in S}\beta_s I_s(t)+\beta_1\cos(\phi_t)+\beta_2\sin(\phi_t)+\sum_{s\in S}I_s(t)\,[\beta_{s,1}\cos(\phi_t)+\beta_{s,2}\sin(\phi_t)]+\sum_{k=1}^{K}h_k g_k(H_t)$	Parameters: $\beta_1,\beta_2,\beta_s,\beta_{s,1},\beta_{s,2},\ s\in S;$<br>$h_k, k=1,\dots,K$
SOP, phase, SOP-phase interaction, history	$\sum_{s\in Q}\beta_s I_s(t)+\alpha_1\mathrm{SOP}(t)+\alpha_2\mathrm{SOP}(t)^2+\beta_1\cos(\phi_t)+\beta_2\sin(\phi_t)+[\gamma_1\cos(\phi_t)+\gamma_2\sin(\phi_t)]\mathrm{SOP}(t)+[\gamma_3\cos(\phi_t)+\gamma_4\sin(\phi_t)]\mathrm{SOP}(t)^2+\sum_{k=1}^{K}h_k g_k(H_t)$	Parameters: $\alpha_1,\alpha_2,\beta_1,\beta_2,\beta_s,\ s\in Q;$<br>$\gamma_1,\gamma_2,\gamma_3,\gamma_4; h_k, k=1,\dots,K$

Model inference methods

Point process GLMs have a number of properties that make them useful for the analysis of spindle event data. They can estimate the simultaneous influences of multiple factors, including non-linear influences, with interpretable parameters(2, 4). These models produce convex likelihoods enabling computationally efficient algorithms (e.g., iteratively reweighted least squares (IRLS)) to compute the maximum likelihood estimators for GLM parameters(2, 3, 5, 6). These algorithms are supported by robust software packages in popular statistical platforms and coding languages such as Python, MATLAB, and R. The fitted parameters from maximum likelihood estimation are asymptotically normal, unbiased, and have minimum mean-squared error. Additionally, the Fisher information for the parameters is computed from the IRLS algorithm, which determines the standard errors of the model parameters, and formal statistical tests can be conducted to determine whether any collection of variables significantly influence the event intensity(6).

Point process GLMs also provide well-established tools for assessing model goodness-of-fit. The model deviance explained is an analogue of variance explained in linear regression, which can be used to compare models(3). Another goodness-of-fit tool is based on the time-rescaling theorem, which transforms the observed inter-spindle intervals into the wait times for a homogenous Poisson process if the model is correct(7). After rescaling, Kolmogorov-Smirnov (KS) plots can be used to compare the distribution of inter-spindle times to those predicted by the model. A well-fitted model produces a KS plot closely following a 45-degree line, and everywhere contained within its significance bounds, while deviations outside the bounds suggest model misfit. A KS test compares

the largest deviation between the empirical and model distributions to known critical values for the hypothesis that the model is correct.

Cardinal spline functions:

History dependence was fit with a GLM as a function of history using cardinal spline basis functions, which have been successfully employed to capture temporal patterns in neural spiking activity(8, 9) and sleep respiratory data. In modeling short term spindle dynamics, we set a tension parameter of 0.5, with end points at 0 and 15 seconds, six knots were evenly placed in every 1.5 seconds up to 9 seconds, with another knot at 12 seconds. To capture spindle infraslow structure, the extended model has six additional knots evenly set in every 10 seconds from 15 seconds to 75 seconds, with an end knot at 90 seconds. In this study, a modified cardinal spline function is used to avoid the boundary effects at the end points(8, 10). For a detailed overview of incorporating spline models into a GLM framework, see Sarmashghi et al(8).

History modulation curve summary statistics

To explicitly quantify features of individualized timing properties in a more interpretable manner, we explored a set of summary statistics that allow us to capture the temporal patterns in individuals and across populations. Based on the history modulation curve, as well as its 95% upper confidence bound (UCB) and lower confidence bound (LCB) at each time lag, we define the following summary statistics:

1. Refractory period: The duration after a spindle during which activity is significantly suppressed. It is quantified as the portion of the history modulation curve significantly less than 1 (The first lag where the UCB is greater than or equal to 1).
2. Excitatory period: The duration after the refractory period during which activity is significantly enhanced. It is quantified as the portion of the history modulation curve significantly greater than 1 (The length of the period from when the LCB is first greater than or equal to 1 to when the LCB returns to be less than or equal to 1).
3. Excitatory peak height: The degree to which the excitation is maximally enhanced. It is quantified as the maximum of the rate multiplier in the excitatory period.
4. Excitatory peak time: The lag at which the maximal excitation occurs. This suggests the most probable interval between spindles. It is quantified as the lag corresponding to the excitatory peak height.
5. Infraslow multiplier: This is quantified as the area under the history curve between 40 and 70 seconds, divided by the duration of that period (30 seconds). It reflects the cumulative effect during the infraslow period.

While other statistics, such as the area under the excitatory period, can be useful for describing history features, we show that it highly aligns with the excitatory period and yields identical demographic results (SI Fig. S11).

Preferred phase, coupling magnitude, and confidence interval:

All the models were fit using the GLM package in MATLAB. The maximum likelihood estimator for the preferred phase was computed from the GLM parameter fits. For

example, after fitting a model with phase, the estimated preferred phase is the four-quadrant inverse tangent(11) of $\hat{\beta}_2$ and $\hat{\beta}_1$, Confidence intervals for the preferred phase were computed as follows. Since the fitted model parameters asymptotically follow a multivariate normal distribution (MVN)(2, 3), 10000 random samples of $\hat{\beta}_1$ and $\hat{\beta}_2$ were drawn from $MVN(\begin{bmatrix}\hat{\beta}_1\\ \hat{\beta}_2\end{bmatrix}, \hat{\Sigma})$, where $\hat{\Sigma}$ is the estimated covariance matrix provided by the GLM fit. The estimated preferred phase was computed for each sample and the 2.5th and 97.5th quantiles were computed. The magnitude of the coupling is computed as $\sqrt{\hat{\beta}_1{}^2 + \hat{\beta}_2{}^2}$. The preferred phase, magnitude, and confidence interval for other models that included the SO phase component were derived similarly. In Figure 7c, the mean parameters from the model (SOP, phase, SOP-phase interaction, history) in each age group were used to visualize the mean phase shift curve.

Deviance explained

The mean fractional deviance explained across population is reported in Result section 1.9, which represents the fraction of the total deviance explained by the full model that is explained by each individual factor. We first computed the total deviance reduction $(\mathrm{Dev_{null}} - \mathrm{Dev_{full}})$ by comparing the null model to the full model (stage, phase, history), then computed the deviance reduction for the stage component $(\mathrm{Dev_{null}} - \mathrm{Dev_{stage}})$ by comparing the null model to the model with single stage component. The fractional deviance explained for stage component is defined as $\frac{\mathrm{Dev_{null}} - \mathrm{Dev_{stage}}}{\mathrm{Dev_{null}} - \mathrm{Dev_{full}}}$. Deviance explained by phase and history are computed similarly. Note, when we report the deviance

explained for SOP, it is computed based on a full model (SOP, phase, history) that uses SOP instead of stage.

Synergy index

We adapted a commonly used measure of information independence for neural coding models(12–15), which is defined as the joint information (I_{AB}) provided by two factors, A and B, minus the sum of the information provided by A and B alone ($I_A + I_B$). A normalized synergy measure(12) is $\frac{I_{AB}-(I_A+I_B)}{I_{AB}}$. It ranges from -1 to 1, where -1 indicates A and B provide totally overlapping (redundant) information, 0 indicates A and B are independent, and 1 indicates that while A or B alone provides no information, they jointly convey complete (synergistic) information.

To test whether phase and history interact to influence spindle dynamics, we computed the information as the deviance explained by each model, so that the synergy index was $\frac{(\mathrm{Dev_{null}}-\mathrm{Dev_{phase+history}})-[(\mathrm{Dev_{null}}-\mathrm{Dev_{phase}})+(\mathrm{Dev_{null}}-\mathrm{Dev_{history}})]}{\mathrm{Dev_{null}}-\mathrm{Dev_{phase+history}}}$, where $\mathrm{Dev_{phase}}$ and $\mathrm{Dev_{history}}$ are the deviance of the models that include the phase, and history components alone, respectively, and $\mathrm{Dev_{phase+history}}$ is the deviance from the model that includes both of these factors.

Topological analysis

We conducted an explorative analysis of fast and slow spindles coupled with frontal and central slow oscillations using the Wamsley dataset. Eleven subjects who had simultaneous central (C3) and frontal (F3) recordings were analyzed. This analysis was

performed for both fast and slow spindles detected from central electrodes, with results presented in SI Fig. S9.

Specifically, we modeled fast spindle activity as a function of central SO phase, sleep stage, and the interaction between stage and phase. We then compared this model to one that used frontal SO phase, sleep stage, and stage-phase interaction. By fitting these models, we can compute the coupling phase differences in N2 & N3 stages, as well as the coupling magnitude for each individual. Similarly, we analyzed the coupling differences for slow spindles.

Statistical tests

Likelihood ratio tests were performed to determine whether short/long-term history, phase, stage-dependent phase coupling, and stage-dependent history contributed significantly to spindle activity. Kolmogorov-Smirnov (KS) tests were used to evaluate the goodness-of-fit of the models (Result section 1.9). In Figure 5b, Figure 7c, SI Fig. S4, and SI Fig. S10b, permutation tests with global bounds were performed to compare history curves across different groups(16). t-tests (corrected for multiple comparisons) were conducted in Figure 4b&c and Figure 5a to compare the mean values ($*$, $**$, $***$ denote the p-value <.05, <.01, <.001 separately). P-values from the t-tests assessing the significance of the Pearson correlation coefficients are reported accordingly. In Figure 7a&b, and SI Fig. S5, the Watson-Williams test was performed to test the significance of the preferred phase shift across sleep depth. In SI Fig. S9, one-sample test is used to test if the population has mean angle 0 (circ_mtest, Circular Toolbox(17)). All statistical

analyses were performed in MATLAB_R2022b. Significance levels of 0.05 were used, if not otherwise specified.

Artifact detection

Artifact detection is implemented in the time domain by an iterative procedure described in Stokes et al(18). Briefly, to detect high-frequency artifacts, the raw data is filtered into a high-frequency (35 Hz to Nyquist) component, and then any data outside of the threshold (mean ± 3.5 × standard deviation) is identified as an artifact and removed; To detect noise with broadband energy, the raw data is filtered from 2 Hz to Nyquist, then the same exclusion criteria are applied to mark artifacts. This iterative approach computes a new threshold each time and removes artifacts until no data exceeds the threshold.

Event detection

The automated spindle detection method is based on our previous work(19), which identifies transient oscillation activity using time-frequency peaks in the sigma range of the EEG spectrogram, which are termed TFσ peaks. Traditionally-scored spindles have been shown to be but a subset of the underlying neurophysiological activity within the spindle range of the EEG(18, 19), due to the origins of spindle identification based on time domain identification by eye based on standards from the 1930s(20). This approach has been shown to provide a less biased representation of the spindle activity with increased night-to-night stability and greater statistical power than traditional spindle identification approaches while retaining the equivalent waveform morphology(19). To investigate fast spindle dynamics, we detect TFσ peaks in the frequency range from 12

to 16 Hz, commensurate with the “fast” spindle activity that is primarily studied. The detected peak time-frequency centroids were used as the timing for point process events. Event times were then discretized into 0.1-second intervals, and a binary spindle event train was computed, indicating whether a spindle event (0 or 1) occurred in each time bin. For simplicity, we refer to the events as spindles, but it is vital to note we are using a more robust and principled event basis. Note, slow spindle activity (TFσ peaks in 9 – 12 Hz) is also analyzed as an additional analysis. However, unless otherwise specified, the term "spindles" refers exclusively to fast spindles throughout this paper.

Slow oscillation phase and power

To estimate the slow oscillation phase (SO-phase), the EEG signal was first band passed from 0.4 to 1.5 Hz using a zero-phase filter, and the Hilbert transform of the filtered signal was applied to create the analytic signal. The instantaneous phase was estimated by computing the angle of the analytic signal. TFσ peak phase was computed using linear interpolation of the unwrapped phase at the TFσ peak times (defined by the spectral peak centroids), and all the phase values were re-wrapped to $-\pi$ to π, where phase 0 means the peak of the slow oscillation, $-\pi$ to 0 and 0 to π correspond to rising and falling phase, respectively.

To estimate the slow oscillation power (SO-power), a multitaper spectrogram(21, 22) of the EEG signal after artifact detection was computed with the following parameters: 3 tapers and time-half bandwidth of 2, 4-second window length with 1-second step size. The raw SO-power was then computed by integrating the power in the slow oscillation

frequency range (0.4 – 1.5 Hz) and converting it to decibels (dB). To standardize across participants, the SO-power was normalized by subtracting the 2nd percentile of the raw SO-power in non-wake stages (REM + NREM), which tends to coincide with light or N1 sleep. This acts as an objectively defined point of alignment for comparison across individuals and populations.

Detailed event detection algorithm

This automated spindle detection method is based on our previous work from Dimitrov et al.(19), which extracts and identifies time-frequency peaks in the sigma range of the EEG spectrogram (TFσ peaks). The goal of this approach is to create a method of spindle detection that is not as prone to the amplitude selection biases and rarity assumptions present in traditional spindle detection. In this section, we outline an enhanced version of the approach that demonstrates greater robustness and accuracy. The details of this event extraction are described explicitly in Dimitrov et al. Everything is preserved except for the clustering algorithm, which we will demonstrate in the next section. However, here is an overview of the algorithm.

Our method focuses on analyzing transient oscillatory activity in the time-frequency domain, which appears as salient peaks in the multitaper spectrogram. The parameter settings for the multitaper spectrogram are as follows: a 1-second window with a 0.05-second step size, 3 tapers, and a time-half bandwidth of 2. This algorithm extracts these peaks by searching for regions with distinct peak-like structures in both time and

frequency dimensions. This is done in two steps, using the concept of peak prominence, which measures the height of a peak relative to its local baseline.

In the first step, called the "frequency step," we detect peaks in the EEG power spectrum at each time and estimate the prominence of the largest peak in the spindle frequency range. In the second step, the "time step," we identify temporal peaks in the time trace of prominence values obtained from the "frequency step." Each temporal peak found in the "time step" therefore corresponds to a well-defined local maximum on the spectrogram. To describe the shape of these time-frequency peaks, we calculate properties such as prominence, duration, central frequency, and bandwidth (quantified in Dimitrov supplementary materials). Before clustering to separate noise peaks, we exclude peaks with durations of less than 0.3 seconds to match established criteria. Additionally, we exclude peaks with frequency bandwidths less than half of the spectral resolution of the spectrograms, as they are not accurately measurable.

The original algorithm used a two-class k-means clustering on the prominence values of all time-frequency local maxima in the 9–17 Hz range. However, this approach is effective only when there is a clear separation between actual signals (i.e., putative TFσ peaks) and noise events. The original algorithm is robust for healthy young adults, where spindles and TFσ peaks are distinct, and signal events have noticeable prominence values that can be separated from the distribution of noise events. Yet, in other groups, particularly older adults where TFσ peaks are less pronounced, the previous method does not perform well because the prominence distribution is unimodal, making the two-class k-means clustering ineffective in automatically identifying TFσ peaks.

To address this challenge and improve the algorithm's general applicability, we analyzed the distributions of four event properties extracted from spectrograms: prominence, central frequency, duration, and bandwidth. Despite variations in the strength of spindles and TFσ peaks, the duration and bandwidth properties consistently exhibit separable peaks at higher values. These properties consistently show triplet peak structures, likely corresponding to the split of noise local maxima during spectral estimation.

Instead of relying on a single two-class k-means clustering on log prominence, which could inaccurately split the large noise peak in half, we now apply two separate three-class clusterings on duration and bandwidth. The cluster centers are seeded at 0 sec, 0.3 sec, and 1 sec for duration, and at 0.5 Hz, 2 Hz, and 3.5 Hz for bandwidth. The final separation of signal and noise is determined by combining the cluster memberships from these two properties. Specifically, a time-frequency local maximum is identified as a TFσ peak if its duration and bandwidth both belong to the largest clusters on the two dimensions. This enhanced method for detecting TFσ peaks offers better distinction between signal and noise and is currently the default setting in this algorithm.

The code for the spindle detection algorithm is available as a component of the DYNAM-O toolbox, available at http://sleepEEG.org.

- **SI Figures**

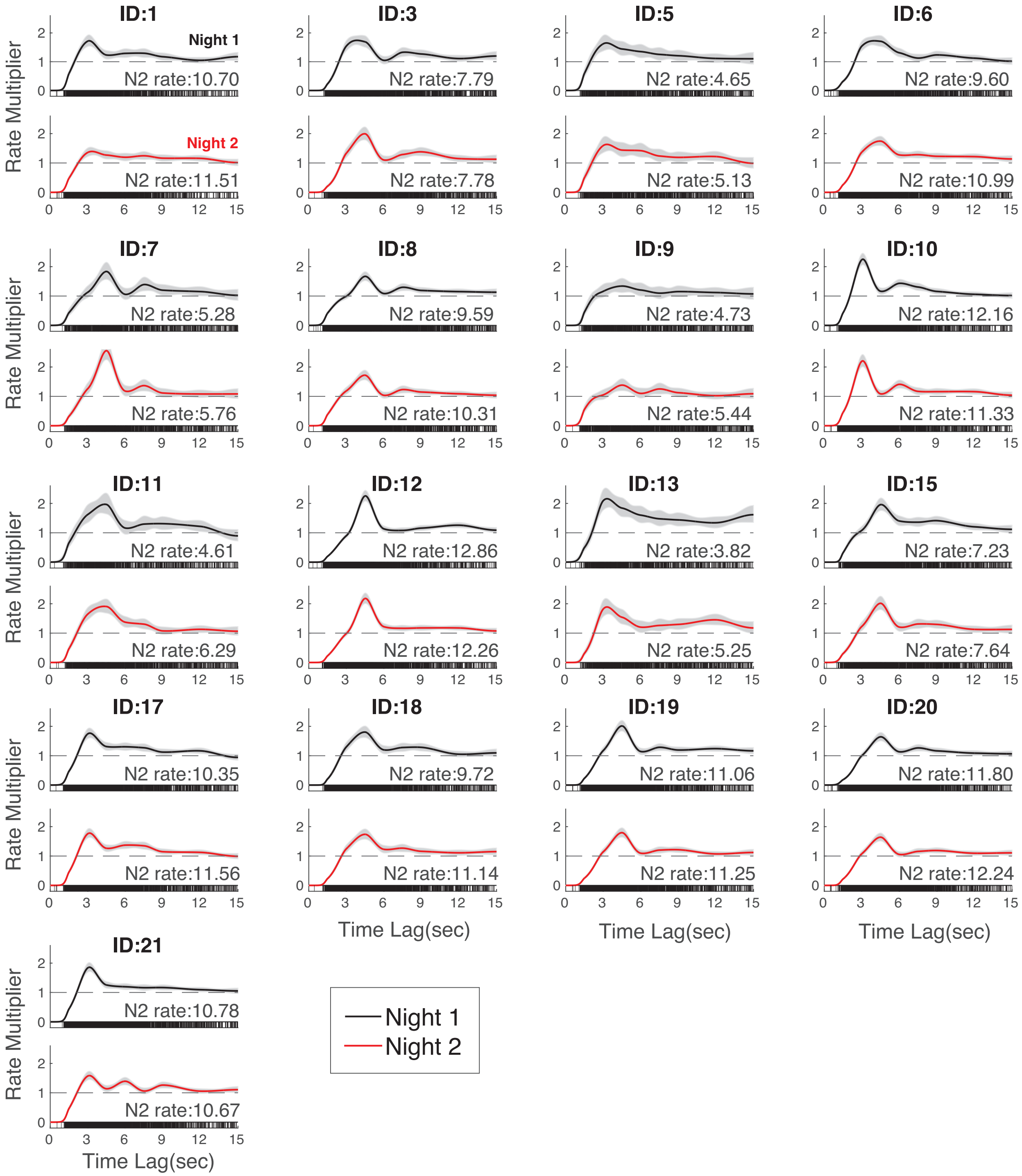

Fig. S1. History dependence exhibits strong night-to-night consistency across all 17 participants in Wamsley dataset.

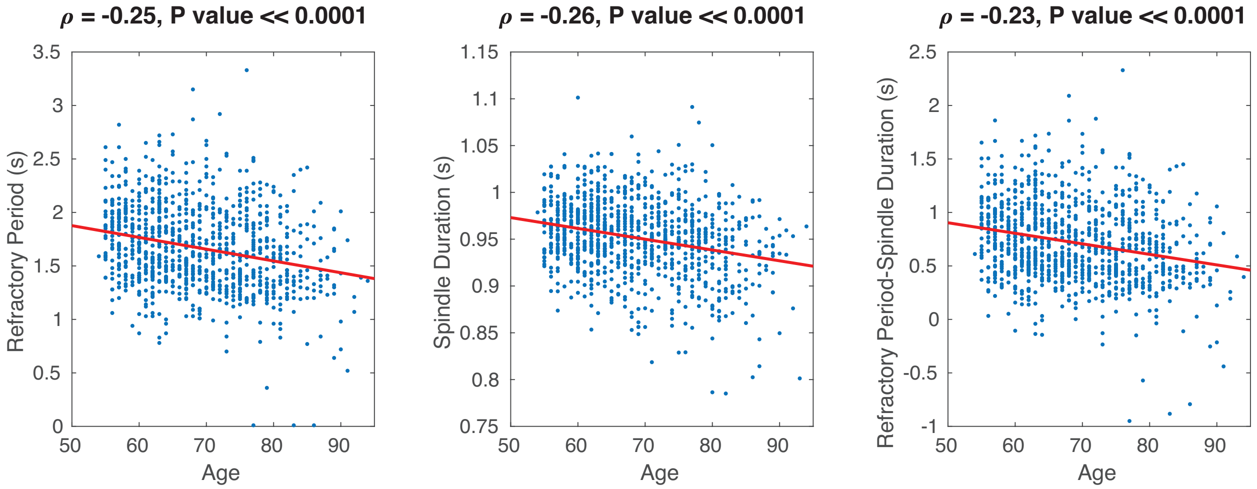

Fig. S2. Scatter plots of refractory period, spindle duration, and refractory period - spindle duration vs. age suggest a reduction in the refractory period with age that cannot be explained by shorter duration spindle events. We show that the refractory period has a negative correlation with age (left panel). One might hypothesize that this relationship could be influenced by spindle duration, which also negatively correlates with age (middle panel). To account for the potential effect of spindle duration, we adjusted the refractory period by subtracting the mean spindle duration for each individual. The adjusted refractory period was then analyzed for its correlation with age (right panel), where we still observe a negative correlation. This suggests that, independent of duration, significant changes in intrinsic spindle timing contribute to a shorter refractory period in older adults. The fitted linear regression lines are shown in red, and correlation coefficients and their P values are reported for each plot.

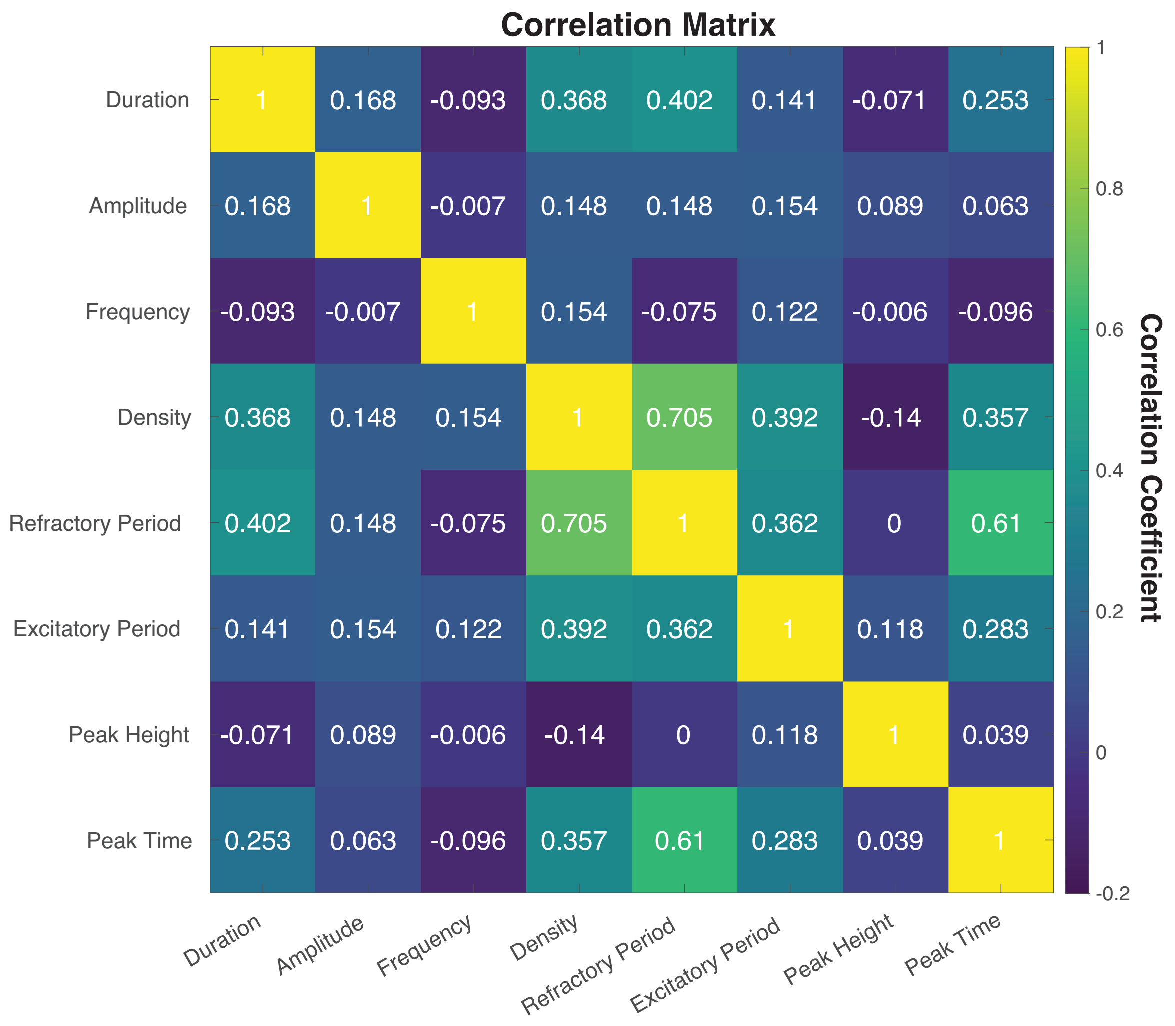

Fig. S3. Mean correlation matrix for spindle history features and spindle morphologies across MESA population.

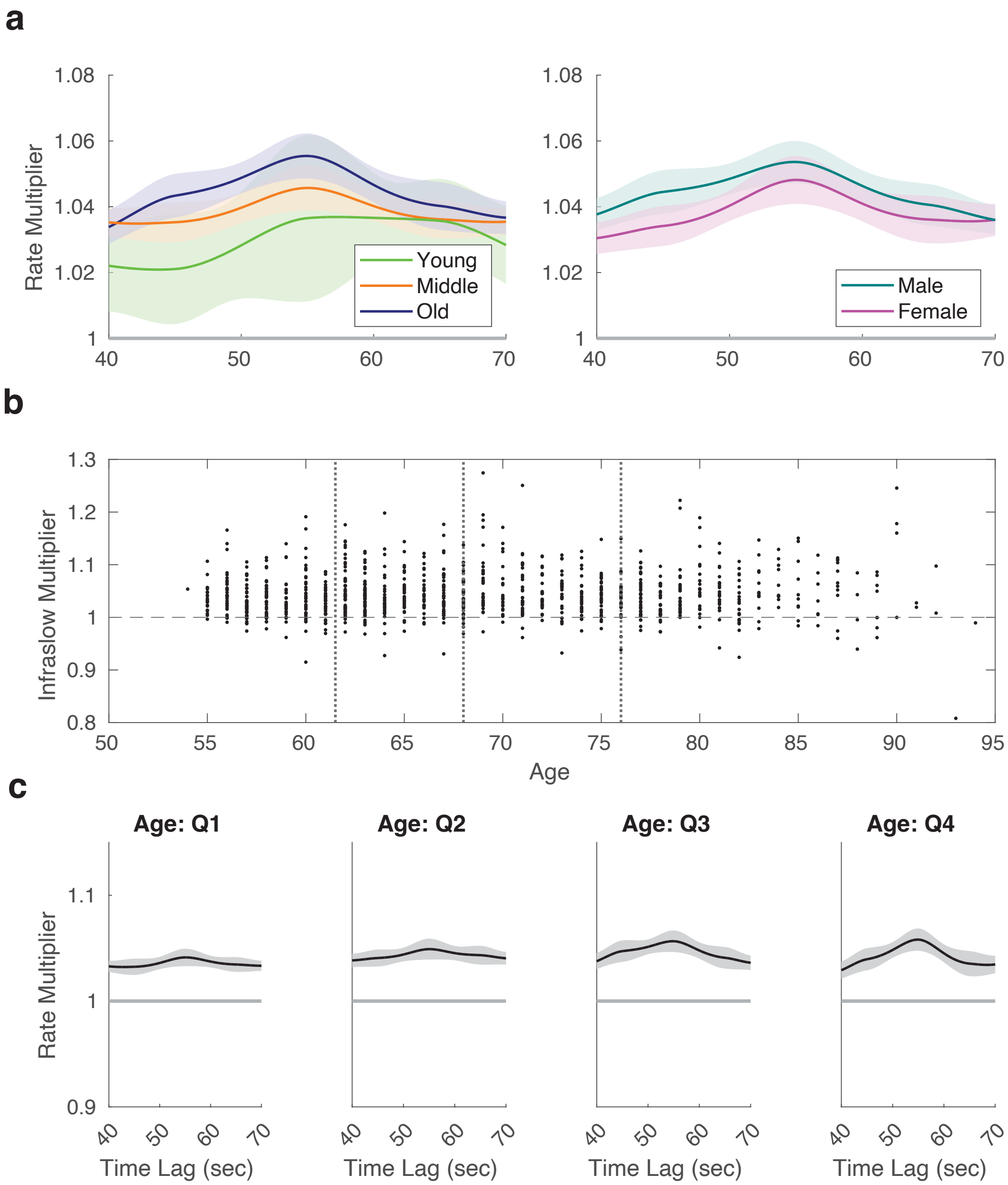

Fig. S4. Demographic differences in spindle long-term history dependence. (a) We extend the analysis to include a 90-second history (covering the infraslow period) and present the mean curves for different age and gender groups. No significant differences in long-term history are observed across all group comparisons. **(b)** In MESA 1008 participants, we observe a small, positive, but non-significant correlation between the infraslow multiplier and age (Pearson's correlation coefficient: 0.059, p value = 0.058). **(c)** We show the average of the history curve for each age quantile group.

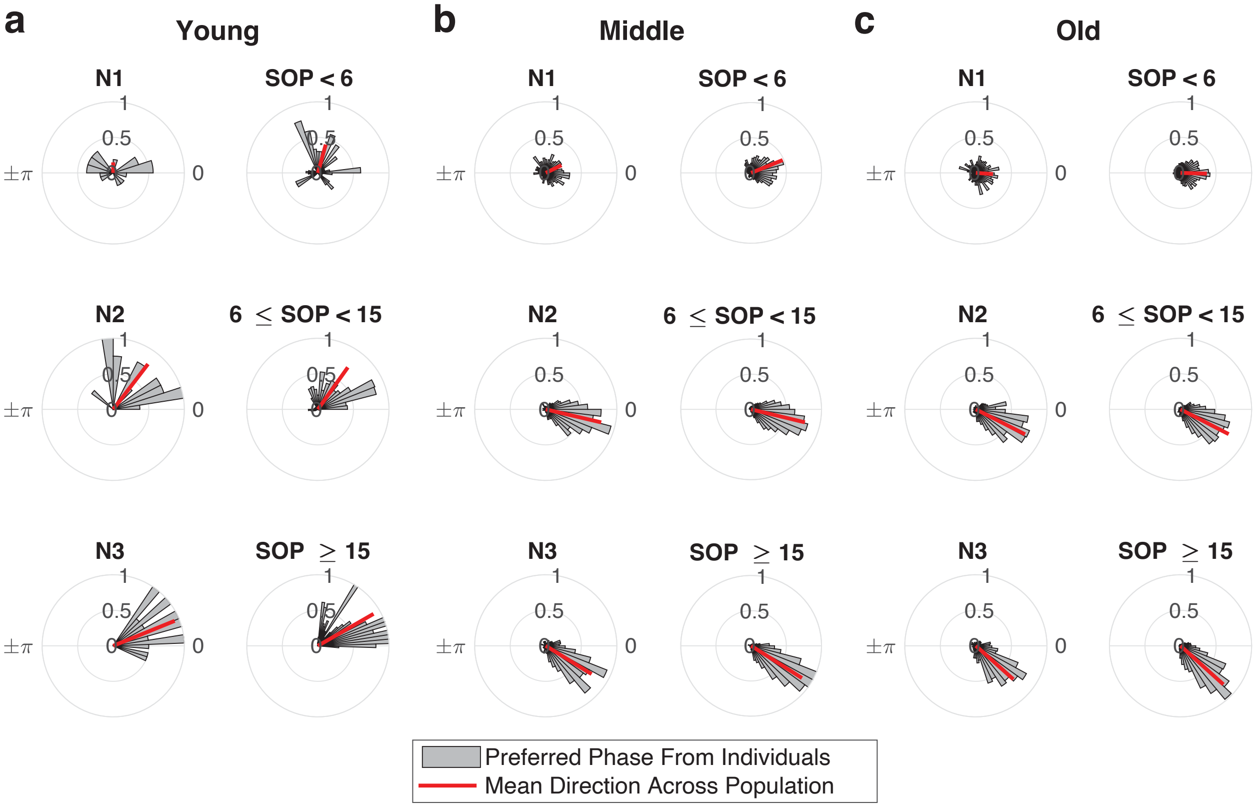

Fig. S5. Phase shift with sleep depth is statistically significant within each age group. Gray polar histograms show the distributions of preferred phase in each group, with the red line showing the mean direction of the polarhistogram. Statistical tests show phase shift is significant within young **(a)**, middle **(b),** and old **(c)** groups(Watson Williams tests, significance level: .05).

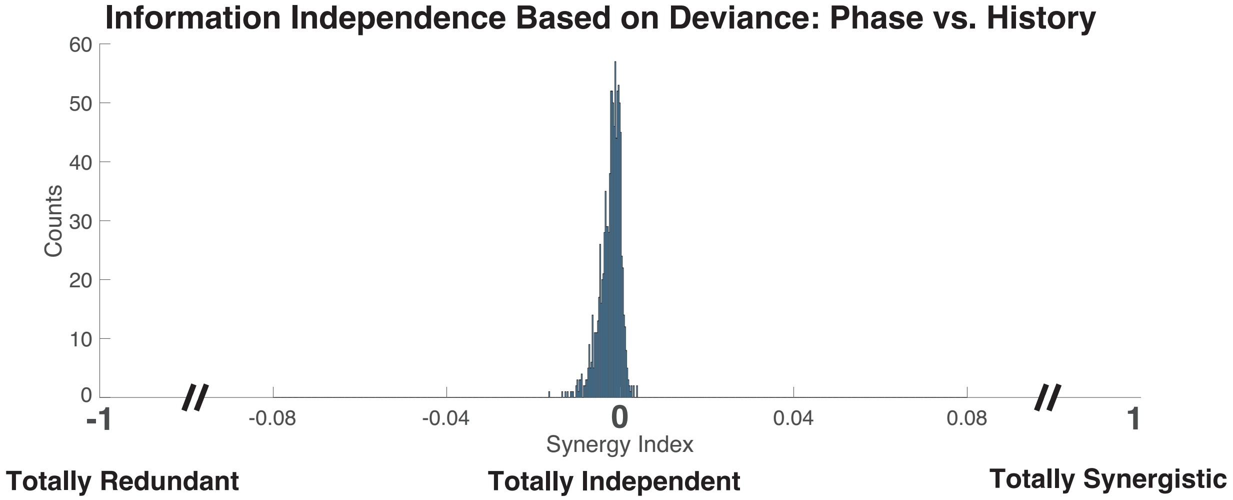

Fig. S6: History dependence and slow oscillation phase provide independent information about spindle dynamics. We show the distribution of synergy index across the MESA population. A value of -1 indicates that two factors provide completely overlapping information (totally redundant), 0 means entirely independent information, and 1 indicates that each component contributes little information on its own, but together they yield substantially more information (totally synergistic). The distribution of the synergy index across the MESA dataset is concentrated around 0, indicating that history and SO phase provide independent information.

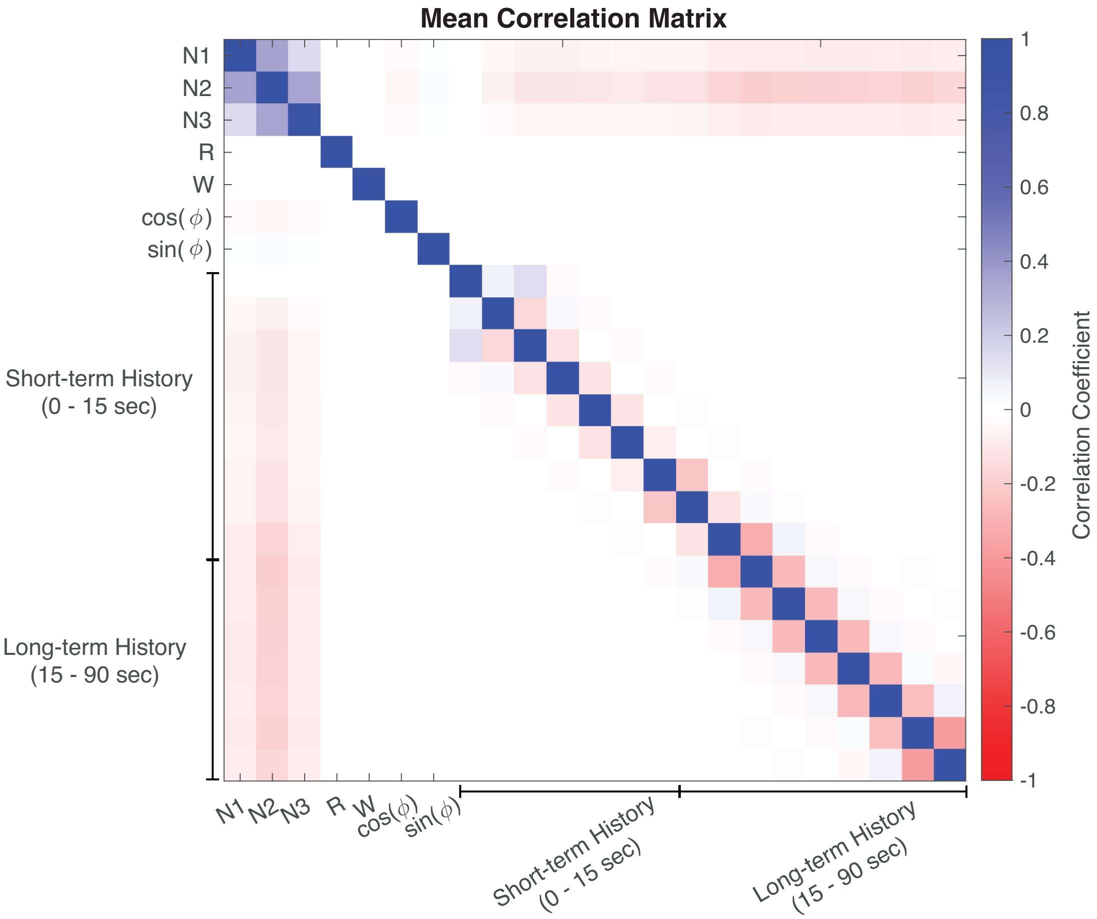

Fig. S7. Mean correlation matrix of modeling components across the MESA population. This matrix represents the mean correlation between the estimated coefficients of different modeling components across the MESA population. We see that phase and history components have almost no correlation with each other, suggesting their independent influences on spindle dynamics. We also observe some correlation between sleep stage and history components, which suggests changing history pattern with sleep depth. Future work can incorporate sleep stage-history interaction term to capture this feature.

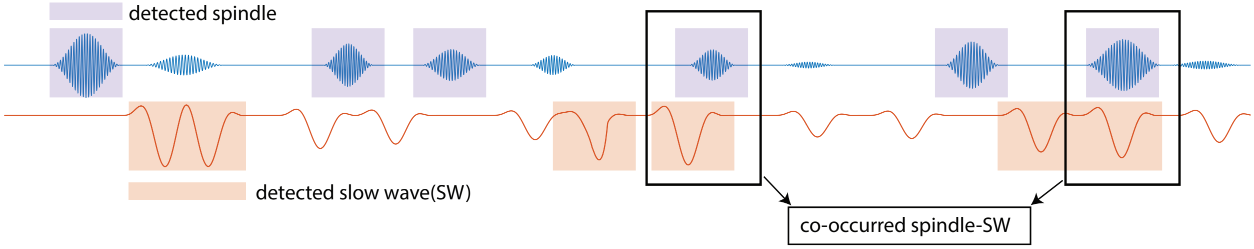

Fig. S8. Spindle/slow wave cooccurrence assumption imposes bias on mechanistic interpretation. The prevailing practices detect spindles and slow waves separately and then select only those that cooccur temporally (black boxes), rejecting all other detected events. Consequently, the average SO/spindle coupling phase is typically computed during the N3 stage, where co-occurrence is more likely. Alternatively, both N2 and N3 stages may be analyzed, but spindles that occur within a specific timeframe of slow waves will be considered. These approaches raise several concerns: First, coupling analysis is most often focused on N3 given the definitional increased prevalence of SOs, yet targets fast spindles, which are most predominant in N2. Furthermore, by discarding all events that do not cooccur, analyses make the implicit assumption that SOs are 100% necessary for spindle production, while also introducing selection bias via both spindle and SO detection.

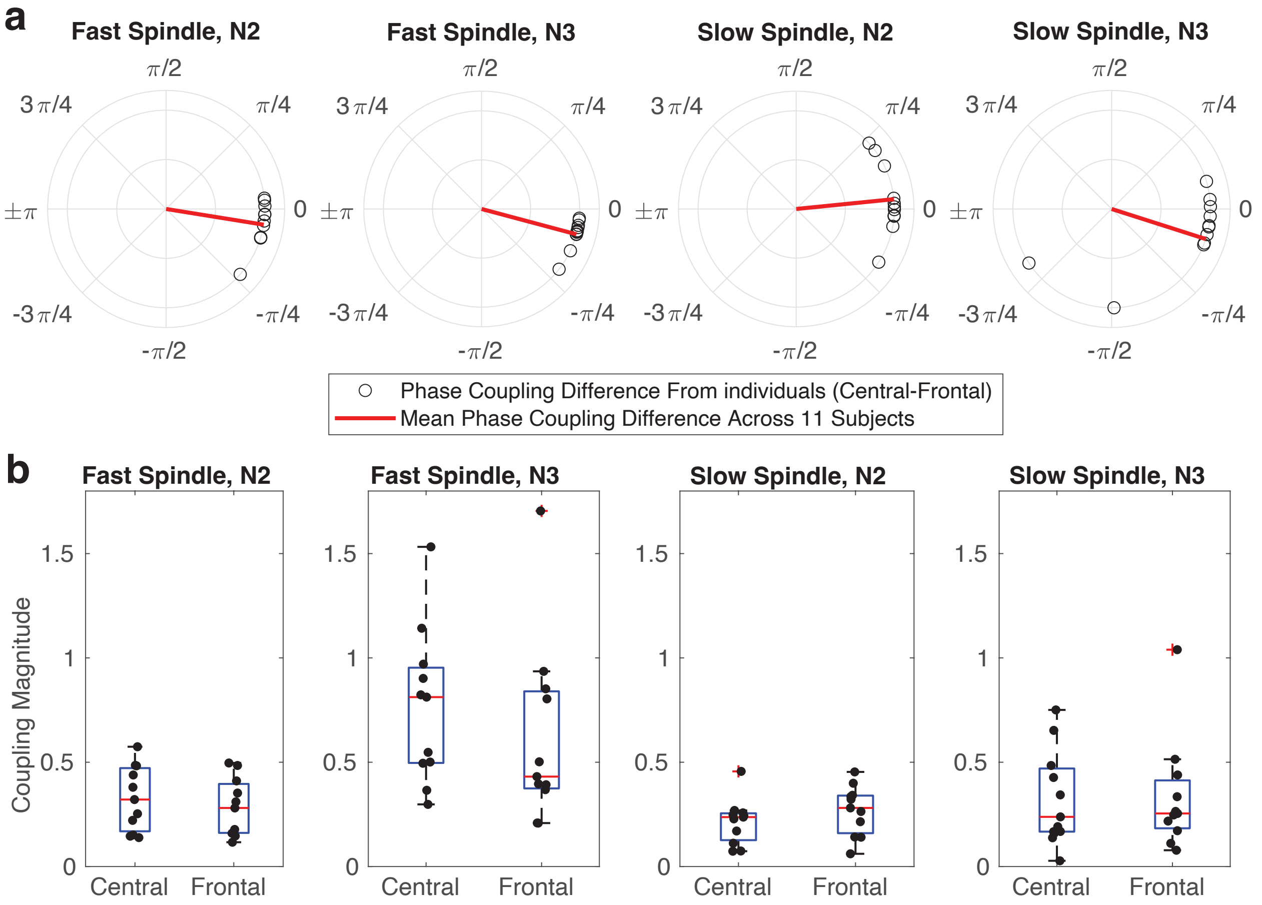

Fig. S9. Topological analysis of stage-dependent SO/spindle coupling. In panel **(a)**, we compare the spindle/central SO coupling to spindle/frontal SO coupling, and show phase coupling differences for fast and slow spindles in N2 and N3 stages. Significant differences are observed for fast spindles, but not for slow spindles (one-sample t-tests for mean 0 angle). Panel **(b)** illustrates the coupling magnitude for each individual, where no significant differences between central and frontal coupling are observed (paired t-tests).

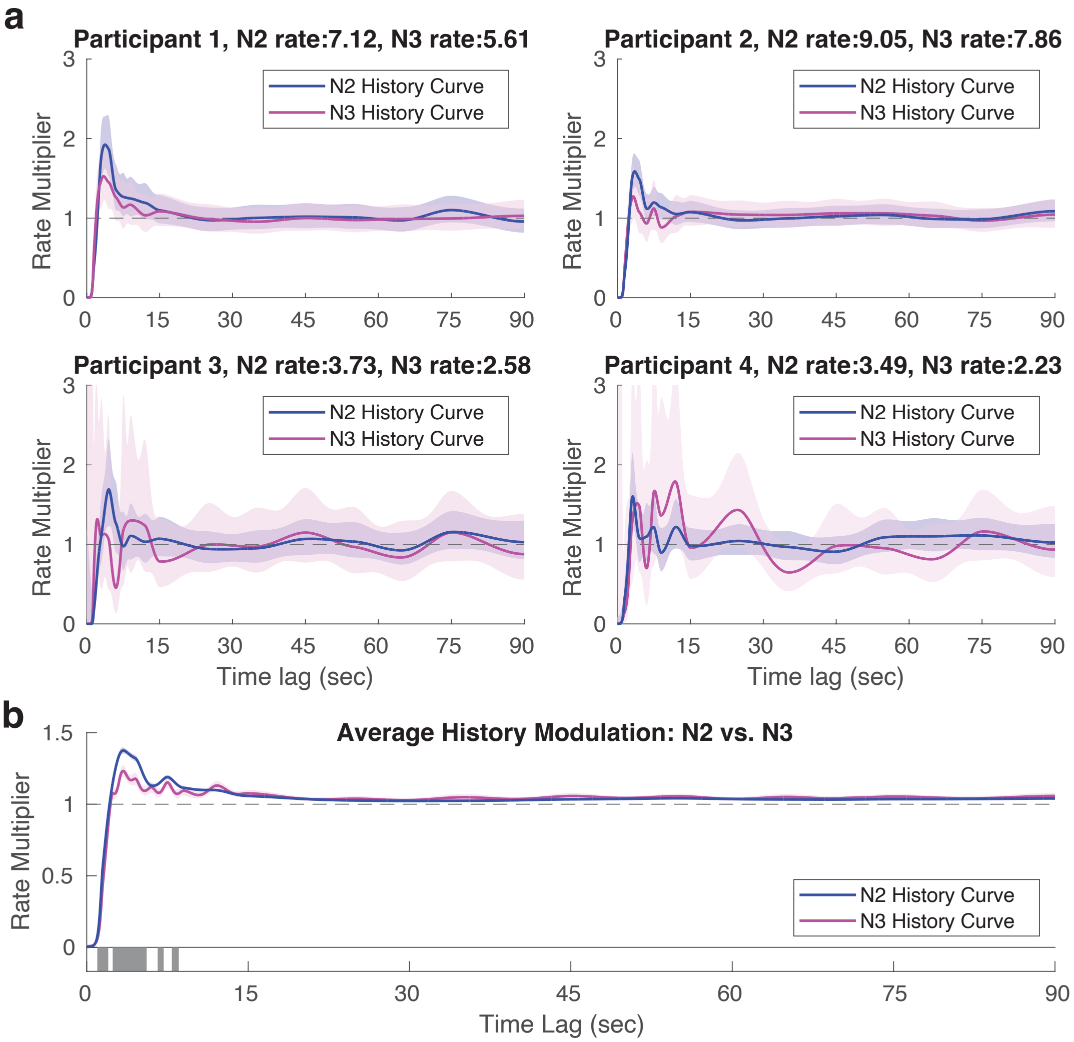

Fig. S10. Stage-dependent history dependence: N2 vs. N3. (a). History curves during N2 (blue) and N3 (magenta) stages are shown for four individuals from MESA datset, shaded with 95% confidence intervals. Participants with higher spindle density often exhibit more confident history curve estimates (top panel, 2 subjects), whereas participants with low N3 spindle rates show extremely large uncertainty in the estimates (bottom panel, 2 subjects). **(b).** The average history curves for N2 and N3 stages across the MESA cohort are displayed, shaded with 95% confidence intervals. Gray blocks at the bottom indicate regions with significant differences using a global permutation test. Note: These average curves represent 826 MESA participants who have a spindle rate of at least 1 events per minute in N3 stage.

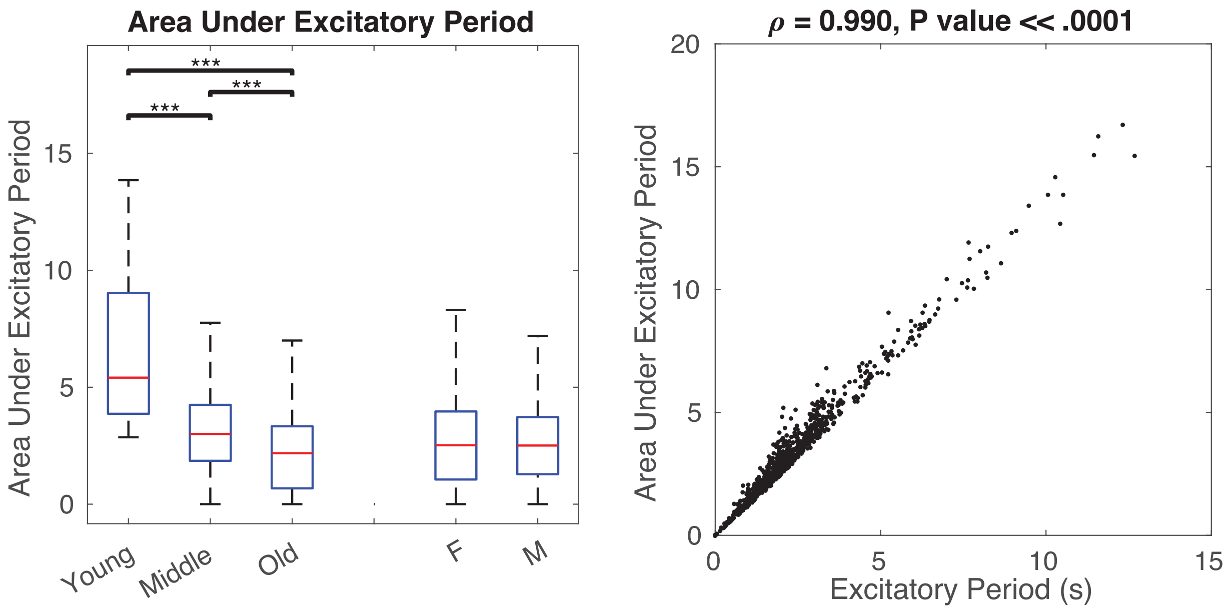

Fig. S11. Area under excitatory period is highly correlated with excitatory period. While other statistics, such as the area under the excitatory period, can be useful for describing features of the history curve, we demonstrate that it closely aligns with the excitatory period and yields identical demographic results.

- **SI References**

1. D. J. Daley, D. Vere-Jones, *An Introduction to the Theory of Point Processes: Volume I: Elementary Theory and Methods* (Springer, 2002).
2. W. Truccolo, U. T. Eden, M. R. Fellows, J. P. Donoghue, E. N. Brown, A point process framework for relating neural spiking activity to spiking history, neural ensemble, and extrinsic covariate effects. *J. Neurophysiol.* **93**, 1074–1089 (2005).
3. P. McCullagh, J. A. Nelder, *Generalized Linear Models*, 2nd Ed. (Routledge, 2019).
4. M. A. Kramer, U. T. Eden, *Case Studies in Neural Data Analysis: A Guide for the Practicing Neuroscientist* (MIT Press, 2016).
5. T. J. Santner, D. E. Duffy, *The Statistical Analysis of Discrete Data* (Springer Science & Business Media, 2012).
6. Y. Pawitan, *In All Likelihood: Statistical Modelling and Inference Using Likelihood* (OUP Oxford, 2001).
7. E. N. Brown, R. Barbieri, V. Ventura, R. E. Kass, L. M. Frank, The time-rescaling theorem and its application to neural spike train data analysis. *Neural Comput.* **14**, 325–346 (2002).
8. M. Sarmashghi, S. P. Jadhav, U. Eden, Efficient spline regression for neural spiking data. *PLOS ONE* **16**, e0258321 (2021).
9. U. T. Eden, R. Amirnovin, E. N. Eskandar, Using point process models to describe rhythmic spiking in the subthalamic nucleus of Parkinson's patients in *2011 Annual International Conference of the IEEE Engineering in Medicine and Biology Society*, (2011), pp. 757–760.
10. S. Chen, S. Redline, U. T. Eden, M. J. Prerau, Dynamic Models of Obstructive Sleep Apnea Provide Robust Prediction of Respiratory Event Timing and a Statistical Framework for Phenotype Exploration. *Sleep* zsac189 (2022). https://doi.org/10.1093/sleep/zsac189.
11. K. Q. Lepage, *et al.*, A procedure for testing across-condition rhythmic spike-field association change. *J. Neurosci. Methods* **213**, 43–62 (2013).
12. E. Schneidman, W. Bialek, M. J. Berry, Synergy, Redundancy, and Independence in Population Codes. *J. Neurosci.* **23**, 11539–11553 (2003).
13. N. S. Narayanan, E. Y. Kimchi, M. Laubach, Redundancy and Synergy of Neuronal Ensembles in Motor Cortex. *J. Neurosci.* **25**, 4207–4216 (2005).

14. C. E. Shannon, A mathematical theory of communication. *Bell Syst. Tech. J.* **27**, 379–423 (1948).

15. N. Brenner, S. P. Strong, R. Koberle, W. Bialek, R. R. de Ruyter van Steveninck, Synergy in a neural code. *Neural Comput.* **12**, 1531–1552 (2000).

16. S. Fujisawa, A. Amarasingham, M. T. Harrison, G. Buzsáki, Behavior-dependent short-term assembly dynamics in the medial prefrontal cortex. *Nat. Neurosci.* **11**, 823–833 (2008).

17. P. Berens, CircStat: A MATLAB Toolbox for Circular Statistics. *J. Stat. Softw.* **31**, 1–21 (2009).

18. P. A. Stokes, *et al.*, Transient oscillation dynamics during sleep provide a robust basis for electroencephalographic phenotyping and biomarker identification. *Sleep* **46**, zsac223 (2023).

19. T. Dimitrov, M. He, R. Stickgold, M. J. Prerau, Sleep spindles comprise a subset of a broader class of electroencephalogram events. *Sleep* (2021). https://doi.org/10.1093/sleep/zsab099.

20. A. L. Loomis, E. N. Harvey, G. Hobart, Potential rhythms of the cerebral cortex during sleep. *Science* **81**, 597–598 (1935).

21. D. J. Thomson, Spectrum estimation and harmonic analysis. *Proc. IEEE* **70**, 1055–1096 (1982).

22. M. J. Prerau, R. E. Brown, M. T. Bianchi, J. M. Ellenbogen, P. L. Purdon, Sleep neurophysiological dynamics through the lens of multitaper spectral analysis. *Physiology* **32**, 60–92 (2017).
